# Cardiovascular Risk Assessment in Patients with Rheumatoid Arthritis

**DOI:** 10.3390/jcm14186461

**Published:** 2025-09-13

**Authors:** Ruxandra Oiegar, Dana Pop

**Affiliations:** 1 4th Department of Internal Medicine, Department of Cardiology Rehabilitation, “Iuliu Hațieganu” University of Medicine and Pharmacy, 400012 Cluj-Napoca, Romania; oiegar.ruxandra@elearn.umfcluj.ro; 2 Doctoral School, “Iuliu Hațieganu” University of Medicine and Pharmacy, 400012 Cluj-Napoca, Romania; 3 Department of Cardiology Rehabilitation, Clinical Rehabilitation Hospital, 400437 Cluj-Napoca, Romania

**Keywords:** rheumatoid arthritis, cardiovascular risk, lipid paradox, inflammation, microvascular coronary disease, carotid ultrasound

## Abstract

**Background/Objectives:** Rheumatoid arthritis (RA) is a chronic inflammatory disease characterized by inflammation of the synovium. The inflammation accelerates the development and progression of atherosclerosis, a key phenomenon in the onset of cardiovascular diseases. The aim of this review was to synthetize the traditional and RA-specific cardiovascular risk (CVR) factors and the CVR assessment guidelines in RA patients. **Methods:** We performed a PubMed search using specific keywords. We synthetized the main findings. **Results:** Although the risk factors are the traditional ones, with certain particularities, the mechanisms that lead to cardiovascular disease are distinguished. In RA, the “lipid paradox” occurs: low levels of total cholesterol and low-density lipoprotein (LDL)-cholesterol, and high levels of high-density lipoprotein (HDL)-cholesterol. Despite this phenomenon, patients have an elevated risk of cardiovascular events. This is due to inflammation, which increases cholesterol catabolism and interferes with the anti-oxidant properties of HDL-cholesterol. There is a significant association between serum C-reactive protein (CRP) value and cardiovascular risk: each 20 mg/L increase in CRP causes a 1% increase in cardiovascular risk. The evaluation of the CVR through standard matrices undervalues the risk in patients with RA. Various approaches have been suggested to improve the accuracy of cardiovascular risk appraisal: from multiplying standard scores, including specific biomarkers, to modifying the impact of certain parameters in risk calculation. **Conclusions:** RA inflammatory and autoimmune mechanisms increase the cardiovascular morbidity and mortality in this group of patients. Therefore, this category of patients requires a proper cardiovascular (CV) evaluation. Carotid ultrasound ensures a better classification of RA patients, especially women, in the cardiovascular risk categories.

## 1. Introduction

Rheumatoid arthritis (RA) is a chronic inflammatory disease characterized by inflammation of the synovial membrane, with a prevalence of 0.5–1%. It occurs relatively frequently, it has an autoimmune mechanism, and its pathogenesis is based on the interaction between genes and environmental factors, with activation of both innate and acquired immune responses. The characteristic chronic inflammation is mediated by cytokines, which are found in significant amounts (interleukins IL-1, IL-6, IL-8, and IL-17, and tumor necrosis factor TNF-α). This abundance of pro-inflammatory cytokines, together with high levels of C-reactive protein (CRP), accelerates the development and progression of atherosclerosis, a key phenomenon in the onset of cardiovascular diseases (CVD) [1].

Although it is a disease that primarily affects the joints, persistent inflammation manifests throughout the body, including the cardiovascular (CV) system, and thus it is associated with an increased cardiovascular risk. One of the leading causes of morbidity and mortality in RA patients is represented by cardiovascular events. Initially, the increased cardiovascular risk was attributed to traditional risk factors, such as high blood pressure, smoking, dyslipidemia, obesity, and diabetes, which are common in this category of patients. The morbidity and mortality associated with rheumatoid arthritis reduce life expectancy by 5–18 years; this is mainly due to the augmented cardiovascular risk. Cardiovascular damage increases mortality by 45–60% compared to the general population. Although the traditional risk factors’ contribution to the cardiovascular risk (CVR) is significant, the mechanisms that lead to cardiovascular disease are distinguished [1,2,3].

In this context, we aim to analyze how cardiovascular risk can be assessed in RA patients. We performed a PubMed search using keywords such as rheumatoid arthritis, cardiovascular risk, inflammation, C-reactive protein, interleukin-6, microvascular disease, and carotid ultrasound. The research period was between March and August 2025. The inclusion criteria involved articles published no sooner than 2000 (with a main focus on articles published after 2010), articles centered around the pathogenic mechanisms of RA, the cardiovascular complications of RA, and the evaluation of the cardiovascular risk in RA. The exclusion criteria comprised articles published before 2000 and articles focused on other rheumatic diseases (lupus, psoriasis, and ankylosing (spondylitis) or on other complications of RA. For synthesis, the studies were grouped based on their main topic: traditional CV risk factors, specific CV risk factors in RA patients, microvascular atherosclerotic cardiac disease in the RA population, CVR assessment in RA, and carotid ultrasound applications in RA.

## 2. Traditional Cardiovascular Risk Factors

Recent studies have suggested that in the absence of established cardiovascular risk factors, RA patients would not have an increased cardiovascular risk. Similarly to the general population, RA patients have traditional modifiable and non-modifiable cardiovascular risk factors. The first category includes smoking, high blood pressure, dyslipidemia, obesity, insulin resistance, and a sedentary lifestyle, while the second category includes gender, age, race, and family history. The modifiable risk factors may occur even more often in the affected group than in the unaffected population. However, the excessive cardiovascular risk of RA patients cannot be explained by the mere presence of these classic risk factors. The persistent inflammation in RA leads to an accentuated effect of the modifiable risk factors, which translates into a subsequent increase in cardiovascular risk [1,2]. In the following part, classic CVR factors will be discussed, focusing on their particularities related to the RA population.

### 2.1. Age

Age is a traditional cardiovascular risk factor. The onset of rheumatoid arthritis at an older age is associated with a higher cardiovascular risk, with a much faster rate of increase in carotid intima–media thickness. This may be caused by a change in the lipid profile (which becomes pro-atherogenic) and the development of high blood pressure with age. In addition, inflammation (and thus the serum CRP levels) is much more pronounced in elders [4].

Although the cardiovascular risk traditionally increases with age, in the case of patients with RA, it should also be appropriately assessed in younger patients. In Rochrich’s study [5], a statistically significant difference between predicted and observed CVR was observed in patients under 55 years of age (5.3% vs. 8%, *p* < 0.001). Approximately 30% of cardiovascular events recorded during follow-up occurred in young patients (<55 years), considered to have a low CVR. The early onset of cardiovascular events may be explained by the dyslipidemia and inflammation present during periods of rheumatic disease activity, thus requiring a shorter period of time for atherosclerotic plaque development and the onset of instability/rupture [5].

Some studies suggest that cardiovascular risk is miscalculated in young subjects and women. SCORE grids tend to underestimate the CVR in patients with RA, especially in those considered at low-intermediate risk. Young patients with RA have the highest relative risk (compared to age-matched healthy subjects), with respect to the relative risk of elderly patients with RA (in relation to age-matched non-RA subjects, with the relative risk of CVR increasing with age) [5].

### 2.2. Gender

RA predominantly affects women, with a 3:1 ratio in their favor. Women with RA are at higher cardiovascular risk. Early menopause (resulting in a reduction in the cardio-protective effect of estrogen) may be, on one side, a predictor of rheumatoid arthritis and, on the other side, it may be caused by it (due to systemic inflammation) [1,5].

Rochrich et al. [5] followed a cohort of patients with RA for 10 years. In the female group, the majority of subjects (59%) had a low predicted cardiovascular risk, whereas in the male group, 68% of subjects had an intermediate/high predicted cardiovascular risk. A statistically significant difference between the predicted and observed CVR was observed in the whole cohort, but this difference was more evident in the female group. In addition, among women with RA who were classified as having a low CVR (<10%), 36% subsequently developed cardiovascular disease. This phenomenon was observed in only 10% of men with RA who had a low predicted CVR. The results of this study emphasize the tremendous role gender plays in CVR estimation. This aspect needs to be taken into consideration, especially in RA, a disease predominantly affecting the female gender [5].

Gender influences different metabolic pathways within the organism (including lipid metabolism), with pathophysiological and clinical cardiovascular consequences in RA patients. These pathways will be discussed in further sections of this review.

### 2.3. Dyslipidemia

In patients with RA, the so-called “lipid paradox” occurs, which involves a reversal of the lipid balance: low levels of total cholesterol and low-density lipoprotein (LDL)-cholesterol, and high levels of high-density lipoprotein (HDL)-cholesterol. Despite this phenomenon, patients with RA are at heightened risk of cardiovascular events [1].

The mechanism of this phenomenon is not well established. Some studies have emphasized the role of inflammation in this process by stimulating cholesterol catabolism and interfering with the anti-oxidant properties of HDL-cholesterol. IL-6 and TNF-α are responsible for the increased number of LDL and SR-B1 (scavenger receptor, class B type 1) receptors in the hepatocyte membrane, resulting in the removal of cholesterol from the plasma and its increased excretion into the bile. Continuous inflammation, on the other hand, stimulates the oxidative metabolism of cholesterol molecules. This oxidized cholesterol becomes antigenic and causes endothelial injury and dysfunction, the process that initiates atherosclerosis. The build-up of oxidized lipids and lesions in the intima creates a vicious circle, in which inflammation is maintained by the continuous release of pro-inflammatory cytokines (TNF-α or IL-1), oxygen and nitrogen radical formation, and tissue damage. HDL-cholesterol plays an important role in slowing these processes; however, its protective actions are interrupted by the systemic inflammation in RA [1].

In patients with RA, there are changes in the (apolipoprotein B) ApoB: (apolipoprotein A-I) ApoA-I ratio, caused by the increase in ApoB levels (with pro-atherogenic properties) and the decrease in ApoA-I levels (with an anti-atherogenic profile). The increase in this ratio, together with the increase in the atherogenic index (high total cholesterol:HDL cholesterol ratio) and the decrease in total cholesterol, LDL-cholesterol, and HDL-cholesterol values, contribute to the pro-atherogenic dyslipidemia of this category of patients. ApoB exacerbates inflammation in RA by binding to immune cells and increasing the release of pro-inflammatory cytokines (IL-1β, IL-6, and TNF-α) [6,7].

Previous studies suggest that lipid metabolism in women and men diagnosed with RA is not similarly affected by systemic inflammation. The HDL-2 fraction is low in women. HLD cholesterol is made up of several fractions: HDL2-c and HDL3-c are responsible for the anti-atherogenic effects of this lipoprotein. Inflammation lowers the level of serum HDL cholesterol and impairs its cardio-protective quality. Therefore, the excessive cardiovascular risk in women with RA may also be explained by changes in the HDL-c fractions (qualitative alteration), in addition to its quantitative reduction [5,8].

In the general population, the cardio-protective effect of HDL-c appears to be the strongest in women. Inflammation decreases the anti-oxidative capacity of HDL, it prevents retrograde cholesterol transport, and it even causes the HDL profile to change from anti-inflammatory to pro-inflammatory. The HDL-2 fraction is responsible for the anti-atherogenic effect of HDL-c. Its higher susceptibility to microenvironment changes (as seen in chronic inflammatory diseases) explains the loss of HDL-c function in the RA population [8].

Rheumatologic disease activity also contributes to reduced levels of HDL2-c. The concentration of this fraction decreases by 0.06 mmol/L for each one-point increase in DAS28 (*p* = 0.05, DAS28 = Disease Activity Score in 28 joints). The serum level of HDL3-c does not appear to be influenced by rheumatologic disease activity [8].

Thus, low levels of serum HDL2-c contribute to the increased cardiovascular risk in women with RA. In these cases, the HDL function is altered, even leading to a change in the anti-atherogenic quality of this molecule to pro-atherogenic. Reduction in the HDL2-c/HDL3-c ratio results in the prevention of retrograde cholesterol transport, a mechanism that is imperative in preventing the development and progression of atherosclerosis. Compared to the general population, mortality is higher in patients with RA, both women and men; however, cardiac mortality is higher in women with RA compared to men with RA due to these variations in lipid metabolism [8].

### 2.4. Smoking

Bedeković et al. [2] observed in their study, which compared CVR between patients with RA and arthritis in the Croatian population, a higher prevalence of smoking in RA patients (52.4%) compared to the general population (31.1%). In fact, smoking is also a risk factor in the development of rheumatoid arthritis. The risk of developing rheumatoid arthritis is twice as high in male smokers and 1.3 times higher in female smokers. Even short periods of smoking are involved in the development of rheumatoid arthritis [2,9].

Smoking increases oxidative stress in RA patients through the alteration of the body’s anti-oxidant mechanisms by free radicals (which are also involved in the pathogenesis of RA). Through its action on cellular and humoral immunity, smoking causes an elevated pro-inflammatory status. It also stimulates the increased synthesis of cytokines and other pro-inflammatory molecules such as fibrinogen, CRP, ICAM-1 (inter-cellular adhesion molecule–1, a transmembrane protein involved in cell to cell adhesion and leukocyte migration), E-selectin (a protein involved in leukocyte recruitment during inflammation), TNF-α, IL-1, IL-6, and IL-17, which are involved in the development and progression of both RA and atherosclerosis [9].

### 2.5. Diabetes Mellitus

Patients with RA have a 1.5-fold higher incidence of type 2 diabetes mellitus compared to the general population. In the same study by Bedeković [2], researchers found a 4-fold higher prevalence of diabetes in RA patients (32.9%) compared to the general population (8.3%).

In patients with RA, abnormal glucose response and insulin resistance are frequent, making RA a risk factor for T2DM (type 2 diabetes mellitus), according to previous studies. The onset and the progression of diabetes mellitus (DM) are facilitated by chronic inflammation. Fasting glucose and insulin levels are elevated in patients with RA. A higher disease activity score (DAS28) entails a significantly higher incidence of DM (odds ratio OR = 75.22, 95% CI: 23.33–242.72, *p* < 0.0001, CI—confidence interval) [10].

Various cytokines, including IL-1, IL-6, and TNF-α, exert their influence on glucose and insulin homeostasis. TNF-α regulates the insulin signaling pathway, aiding in the development of insulin resistance. It lowers the secretion of insulin and suppresses organ sensitivity to it. The insulin resistance also results from elevated serum levels of CRP, characteristic of this population of individuals. Insulin resistance is an individual prognostic factor in patients with RA, indicative of subclinical atherosclerosis, which can be quantified by carotid intima–media thickness. The implications of TNF-α in the onset of DM in RA patients are supported by the effects of TNF-inhibiting treatment on insulin resistance and glucose metabolism. TNF inhibitors proved effective in reducing HbA1c levels even after only one month of treatment, supporting the involvement of this cytokine in glucose metabolism [6,7,11,12,13].

On the other hand, hyperglycemia negatively impacts the immune cells, leading to a hyperproduction of advanced glycated end products and reactive oxygen species, which both stimulate the synthesis of pro-inflammatory cytokines, creating a vicious cycle. Despite the fact that T2DM and RA have similar inflammatory pathological mechanisms, there is limited evidence to support T2DM as a risk factor for RA. According to Lu et al. [14], despite the higher prevalence of T2DM in RA patients rather than in the control group, it seems possible that T2DM increases the risk of RA only in the female population, especially within the younger cohort; the highest odds of developing RA (OR = 3.59, 95% CI: 1.99–6.49) were found in the 20 to 44 years group, at the age with the most outstanding chance of developing RA. The association was the strongest in the case of a short period of time (<4 years) between T2DM diagnosis and RA diagnosis. In contrast, other studies found that T2DM is a significant risk factor for inflammatory arthritis, increasing the risk by 2.5 times [14,15,16,17].

In light of this evidence, there is an interplay of pathogenic mechanisms linking RA and DM, considering that both are chronic diseases that alter the metabolic homeostasis of the organism.

### 2.6. High Blood Pressure

Hypertension holds an important role in CV risk. An increase of 20 mmHg in systolic blood pressure in patients with RA leads to frequent CV events. The prevalence of this risk factor is even higher in this population. Patients with RA have higher blood pressure values than the general population, high night-time systolic values, and a higher prevalence of resistant hypertension. The pathological causes of this phenomenon include chronic inflammatory aggression on the endothelium, physical inactivity, and genetic factors [7,18].

High levels of CRP determine a reduction in endothelial nitric oxide (NO) production and trigger platelet activation and secretion of vasoconstrictive substances, leading to hypertension. Pro-inflammatory cytokines maintain the elevated blood pressure through endothelial proliferation. In contrast, IGF-1 (insulin-like growth factor 1) supports the endothelial cell function through interaction with its endothelial cell membrane receptor. This mediator promotes blood vessel repair and lowers the impact of oxidative stress on the endothelium. Its vasodilatory properties help in regulating blood pressure, lowering the CVR. The IGF-1/IGF-1R (receptor of the IGF-1) pathway is also important in joint inflammation. Alteration to this signaling mechanism is responsible for more intense pain perception in RA patients [19,20].

Erlandsson et al. [20] followed a cohort of RA patients with regard to their serum IGF-1 levels and hypertension. The authors observed an increased estimated CVR in patients with lower levels of IGF-1. Patients in the IGF-1-low group had more pharmaceutical antihypertensive treatment prescribed, in comparison to the IGF-1-high group. The level of serum IGF-1 was the most prominent difference between these groups, supporting the role of this biomarker in keeping blood pressure under control in RA patients. Patients with low IGF-1 levels were more frequently treated with a methotrexate monotherapy, in comparison to the high IGF-1 group (treated with TNF-α inhibitors), indicating uncontrolled levels of inflammation in this subpopulation. Patients with low IGF-1 levels had an almost 5 times higher risk of cardiovascular events in the future. During the follow-up period, more patients in the IGF-1-low group had more antihypertensive agents added to their treatment, in comparison to their high IGF-1 counterparts. Therefore, IGF-1 has a tight connection to hypertension in patients with RA, being a possibly valuable predictor for CVD development.

In many RA patients, high blood pressure is also due to the regular use of anti-inflammatory drugs, both steroidal (glucocorticoids) and non-steroidal (non-selective or coxibs). In terms of disease-modifying therapy, there are a few important admonitions about high blood pressure. While leflunomide may cause hypertension, methotrexate is associated with optimal control of blood pressure values and a lower risk of hypertension. Targeted biological or synthetic treatments do not appear to cause elevations in blood pressure [7].

### 2.7. Body Weight

In one study [21], body mass index (BMI) had an important association with RA risk. Both overweight and obese women had a higher chance of developing RA. The risk was even higher for women diagnosed early. In addition, abdominal obesity is more important as a risk factor for RA, in comparison to general obesity (hazard ratio HR = 1.22, 95% CI: 1.06–1.41). After age stratification, RA was positively associated with a higher waist circumference (>88 cm) in female patients younger than 55 years (HR = 1.68, 95% CI: 1.30–2.17). In women >55 years, the same relationship did not reach statistical significance. Obesity itself predisposes to a chronic inflammatory status, increasing the production of a wide range of adipokines and cytokines (IL-1, IL-6, IL-17, and TNF-α), which are involved in the pathophysiology of RA. The abundance of these pro-inflammatory substances represents a trigger for autoimmune responses, including rheumatoid arthritis [17,21].

Visceral fat has been previously associated with the development of various chronic diseases. A recent study [22] investigated the possible association between excessive visceral fat and RA. Patients were divided into two groups: RA and non-RA. RA patients had a higher metabolic score for visceral fat (METS-VF); the calculation formula is thoroughly explained in the methods section of this study. A significant, positive correlation was found between visceral fat metabolism and the onset of RA: the prevalence of the rheumatic disease was 2.3 times higher in patients with METS-VF (*p* < 0.001). The connection was still statistically significant after different variables’ adjustments (OR = 1.50, 95% CI: 1.12–2.00, *p* = 0.022). Through ROC (receiver operating characteristic) analysis, authors underlined that METS-VF is a better predictor for RA appearance (AUC = 0.6620; AUC—area under the curve) compared to BMI (AUC = 0.5828) and waist circumference (AUC = 0.6003). These preliminary results suggest a better performance of this parameter in forecasting the onset of rheumatic disease. It acts as an endocrine organ through over-secretion of cytokines, such as IL-6 and TNF-α, leading to a pro-inflammatory status. An increased metabolism of the visceral fat indicates the possibility of underlying metabolic abnormalities, including insulin resistance, exacerbating the chronic inflammation. This altered metabolism not only contributes to the onset of RA but also to its progression and complications, including the cardiovascular ones. Excessive adipose tissue determines an accelerated clearance of anti-TNF agents, a reduced half-time, and an insufficient serum concentration, resulting in less optimal control of inflammation and, therefore, a higher CVR [22,23,24].

On the contrary, results from other studies support the hypothesis that obese patients with RA have a slower progression to the bone erosions stage (equivalent to advanced disease). The advanced stage of RA is associated with more extra-articular manifestations (including cardiovascular complications). This sets higher BMI as a protective mechanism against progressive joint and bone damage, slowing the natural evolution of RA and the appearance of cardiovascular complications [25].

On the other hand, rheumatoid cachexia is a specific cardiovascular risk factor. This implies a loss of muscle mass while the fat tissue increases, thus the patient maintains the same weight. Excessive proteolysis in muscle tissue is caused by pro-inflammatory cytokines (TNF-α in particular) [6].

## 3. Specific Mechanisms Involved in Cardiovascular Damage in RA

### 3.1. Inflammation

**Figure 1:**
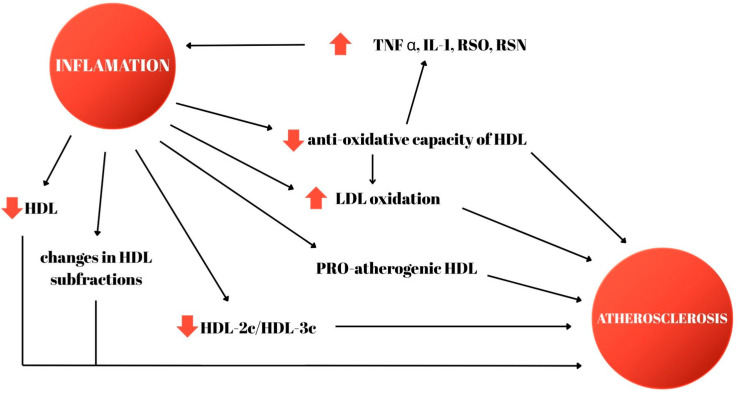
The interaction between inflammation and lipid metabolism (LDL—low density lipids; HDL—high density lipids; TNF-α—tumor necrosis factor-α; IL-1—interleukin 1; RSO—reactive species of oxygen; RSN—reactive species of nitrogen).

Inflammation in RA is a promoter of atherogenesis, and it is responsible for endothelial activation and molecular adhesion, generation of reactive oxygen species, and changes in lipid metabolism, with alterations in lipid particles: increased oxidation of LDL-c and formation of small dense LDL-c molecules (an intensely atherogenic lipid fraction). Elevated serum levels of oxidized lipids, together with RA-specific antibodies, are associated with subclinical markers of atherosclerosis, such as carotid intima–media thickness and coronary calcium score. The accumulation of oxidized LDL lipids in the vascular wall is also precipitated by a decrease in the anti-oxidant capacity of the HDL fraction of cholesterol. Myeloperoxidase, another biomarker with elevated values in patients with rheumatologic pathology, in turn stimulates cholesterol oxidation and foam cell formation, thereby accelerating vascular atherosclerosis. Because of the interdependence relationship between the lipid profile and inflammation, this risk factor should be measured during periods of remission or low rheumatologic disease activity [7]. The complex interaction between inflammation and lipid metabolism is depicted in Figure 1.

Constant inflammation leads to continuous generation of pro-inflammatory cytokines. Activation of T and B lymphocytes induces the formation of antibodies, the best known and most important of which are the rheumatoid factor (RF; an IgM-type antibody that targets the Fc portion of IgG antibodies) and the antibodies to anti-cyclic citrullinated peptides (ACCPs). High titers of these antibodies are responsible for both synovitis and extra-articular manifestations (including cardiovascular damage). In addition, B lymphocytes produce cytokines and chemokines (B-cell activating factor–BAFF, monocyte chemotactic protein 3–CCL7), which are involved in the recruitment of monocytes to the myocardial tissue, causing tissue damage at this level. Neutrophils also play a crucial role in generating and maintaining inflammation in arthritis. These cells generate neutrophil extra-cellular traps (NETs), which maintain the inflammatory response, but have also been shown to be part of the atherosclerotic damage. In addition to the development of atherosclerosis, chronic inflammation also accelerates its progression to plaque rupture and thrombosis [1,26,27].

As regards the CRP, an important marker of inflammation, according to Goodson’s study [28], an increased baseline level > 5 mg/L is associated with all-cause mortality, with HR = 2.7 (95% CI: 0.9–3.0) in men and HR = 2.7 (95% CI: 1.5–5.1) in women, with a strong association with cardiovascular mortality: HR = 3.9 (95% CI: 1.2–13.4) for men and HR = 4.22 (95% CI: 1.4–12.6) for women. According to this study, this association was stronger in patients who met the ACR (American College of Rheumatology) criteria for RA at the time of enrolment in the study, and in HIV (human immunodeficiency virus)-positive patients (in whom a modest increase in CRP levels leads to a 7-fold increase in the cardiovascular risk). However, no effects dependent on the specific value of CRP have been identified.

The baseline CRP level may be predictive of cumulative CRP levels in disease progression (leading to increased cardiovascular risk) and for radiological progression of RA. However, one thing that should also be taken into consideration is that serum CRP levels are also influenced by other traditional risk factors, such as BMI and smoking. Each one-unit increase in CRP caused an increase in cardiovascular risk by 0.8%. Elevated CRP levels > 5 mg/L are also associated with increased vessel reactivity, present in both stable and unstable angina [28].

Erre et al. [29] found in their study a significant association between serum CRP value and cardiovascular risk (as assessed by the ESR-RA score [30]): each 20 mg/L increase in CRP causes a 1% increase in cardiovascular risk. Elevated serum CRP values are also a biomarker for the progression of subclinical atherosclerosis.

Despite a low correlation coefficient between serum CRP levels and cardiovascular risk, this association is significant, as it suggests that residual inflammation may explain a higher baseline for cardiovascular risk in RA patients. Thus, strict control of this persistent inflammatory status can reduce the risk of cardiovascular events [29].

IL-6, another important marker of inflammation, appears to be independently associated with a higher incidence of cardiovascular diseases in RA patients. Roghani’s [31] study provides evidence of an association between elevated serum IL-6 levels and cardiovascular disease. Multiple positive correlations were observed between IL-6 and DAS28, blood pressure, SCORE (Systematic COronary Risk Evaluation), RRS (Reynolds risk score), high-sensitivity CRP, and NTproBNP (N-terminal pro-B natriuretic peptide). Furthermore, a negative correlation with serum HDL cholesterol was also noticed. All these results support the significant role that IL-6 plays in the pathophysiology of cardiovascular disease in RA patients. Thus, this cytokine can be used as a biomarker to assess the potential risk of cardiovascular events in this category of patients. Another study that followed the progress of patients treated with tocilizumab, an IL-6 receptor antagonist, found a significant reduction in cardiovascular risk [31,32].

In conclusion, the effects of inflammatory mechanisms present in RA intensify the atherogenic potential of traditional cardiovascular risk factors: dyslipidemia, smoking, high blood pressure, and diabetes mellitus [1,26].

### 3.2. Rheumatologic Disease Activity

In RA, the CVR is directly proportional to the rheumatologic disease activity: patients with moderate activity have a 35% lower risk than those with high activity, while low activity leads to a 58% reduction in risk. The disease activity can be easily assessed by using DAS28, a score comprising the number of swollen and tender joints, global health (reported on the visual analog scale), and an inflammation marker (either ESR—erythrocyte sedimentation rate—or CRP). One study [33] observed a 7% increase in CVR per acute episode of RA (flare) compared with an appropriate inflammatory status control. Episodes of increased inflammatory activity in RA are associated with increased atherosclerotic plaque vulnerability [7,33,34].

### 3.3. CD163 Overexpression

CD163 is a transmembrane protein found in monocytes and macrophages. The expression of this protein is influenced by pro- and anti-inflammatory cytokines, acute-phase reactants, and the use of corticosteroids. Elevated levels of this membrane receptor and of the serum-soluble form are associated with increased cardiovascular risk. High concentrations of this biomarker are also found in the case of macrophage activation during a flare of synovitis in RA [35].

M2 macrophages, which are constantly present within the joint structure, are responsible for an intense expression of the scavenger receptor CD163, alongside production of both pro- and anti-inflammatory cytokines. The expression of this receptor is upregulated by pro-inflammatory cytokines, such as IL-1 and IL-6. The soluble form of this receptor, sCD163, is a byproduct of TNF-α converting enzyme (TACE), also involved in the release of TNF-α (which holds a major role in the pathophysiology of RA) [36].

Zaragoza-Garcia [35] and colleagues observed elevated serum levels of sCD163 in women with RA and demonstrated the ability of serum sCD163 levels to predict increased cardiovascular risk by observing a positive association with traditional parameters of this risk: age, TC/HDL-c ratio, LDL-c/HDL-c ratio, TG/HDL-c ratio, cardio-metabolic index), and with markers of RA activity—high-sensitive CRP, CHR (high-sensitive CRP: HDL cholesterol ratio), DAS28-ESR (Disease Activity Score in 28 joints, calculated using erythrocyte sedimentation rate), and HAQ-DI score (Health Assessment Questionnaire—Disability Index).

Another study [36] followed patients recently diagnosed with RA, who received treatment with methotrexate and intra-articular betamethasone injections ± cyclosporine. Radiographic measurements were performed at the beginning and periodically after that. The authors compared the serum levels of sCD163 in patients with chronic RA, patients with osteoarthritis (OA), and healthy volunteers. After 9 months of treatment, a mean decrease of 3.3 points in DAS28 was registered. In addition, they found significant differences in the plasma levels of sCD163 before treatment initiation and after 9 months of it. A statistically significant correlation was found between the serum and synovial fluid levels of sCD163 in RA patients. A correlation between sCD163 levels and radiographic changes, over a follow-up period of 5 years, was found. Radiographic alterations indicate a progressive disease, associated with a high inflammation status and, therefore, a higher chance of elevated cardiovascular risk. The association between plasma levels of sCD163 and radiographic progression over the years (if the inflammation and the activity of macrophages are not controlled early in RA) underlines the possible role of this biomarker as a disease progression (and possibly its concomitant complications, including cardiovascular disease) predictor. The link between sCD163 levels and the degree of inflammation is supported by the low levels of this biomarker in patients with OA, a degenerative joint disease.

Previous studies have observed associations of this biomarker with serum levels of CRP and TNF-α. With a plasma half-time of up to 24 h, this biomarker is suitable for serum measurement. These results support the usefulness of sCD163 as a biomarker to estimate cardiovascular risk in women with RA. However, further studies are needed to confirm the role of this biomarker in the proper assessment of cardiovascular risk in patients with rheumatoid arthritis [35,36].

### 3.4. Endothelial Dysfunction

The endothelial dysfunction (ED) tends to appear early in the RA, increasing the risk of atherosclerotic cardiovascular disease. The pathophysiological mechanisms involved in ED are not completely understood, but it is likely that they are not only due to traditional risk factors (such as smoking). Various new metabolic pathways have been proposed as responsible for ED [37].

Endothelial dysfunction is influenced by inflammation and HLA-DR1 (human leukocyte antigen DR1). The degree of endothelial dysfunction can also be assessed by measuring the serum levels of molecules involved in this process, such as VCAM-1 (vascular cell adhesion molecule 1), ICAM-1, and ELAM-1 (endothelial leukocyte adhesion molecule 1/selectin). These molecules play a more important role in the development of cardiovascular disease in RA patients. They show, on one hand, increased synovial inflammation and, on the other hand, exposure of the endothelium to increased serum levels of pro-inflammatory cytokines, with increased dysfunction [38].

Pro-inflammatory cytokines, secreted in the inflamed synovial membrane, impede the normal function of the vascular endothelium both directly and through their effects on insulin sensitivity and the production of CRP and fibrinogen. Rheumatoid factor can also directly cause endothelial injury. The presence of RF, produced by B-type lymphocytes, can independently predict endothelial dysfunction and influence the cardiovascular risk of this category of patients. In contrast to the general population, in which coronary plaques consist almost exclusively of T lymphocytes, considerable amounts of B-cells have been observed in the coronary wall in some RA patients, suggesting the involvement of humoral immunity in the progression of atherosclerosis [27,38].

Humanin, a peptide derived from mitochondria, is a key regulator of the endothelial function, protecting endothelial cells from oxidized LDL (a source of oxidative stress and consequent cell apoptosis). Humanin holds a crucial role in the optimal function of coronary vessel cells. Coradduzza et al. [37] studied the association between this biomarker, humanin, and the endothelial dysfunction in patients with RA. They found that 9-fold higher serum levels of humanin were associated with less endothelial damage, supporting the protective role of this biomarker. In addition, the authors studied the predictability value of humanin for endothelial dysfunction. They obtained an AUC of 0.685 (95% CI: 0.574–0.783, *p* = 0.0015, cut-off level 124.44 pg/mL), indicating a moderate, yet statistically significant, ability of humanin as a predictor of ED. As atherosclerotic CVD, a consequence of ED, increases the mortality and the survival rates, the authors investigated the survival rate of patients with humanin levels above and below the cut-off point (124.44 pg/mL). Patients in the former group were found to have a better survival outcome (*p* < 0.0001). Furthermore, the HR for mortality, based on humanin levels, was 18.66 (95% CI: 6.33–55.02, *p* < 0.0001), underlining the possibility of this peptide as a biomarker for ED and overall survival in RA patients. In light of these discoveries, it is possible that humanin may play an important role in decelerating the atherosclerotic process, thus maintaining the health of the blood vessels, prolonging the development of CVD. Low levels of humanin can indicate, in an early stage, the deterioration of vascular health, leading to CVD.

Another potential biomarker for ED could be ischemia-modified albumin (IMA). Previous studies showed that this chemically altered protein is increased in RA patients, with higher serum levels being attributed to the chronic status of inflammation, oxidative stress, and constant formation of oxygen reactive species. The peripheral vasodilatory function correlated in a significant and inverse manner with the serum levels of IMA (rho = −0.22, *p* = 0.02). RA patients with ED had higher levels of IMA. The serum concentrations of IMA were significantly associated with ED even after the correction of confounding factors [39,40,41].

### 3.5. Drug Therapy

Some of the drugs commonly used to treat RA, such as glucocorticoids, lead to an increase in cardiovascular risk [2].

Cardiovascular risk is also increased by the constant use of anti-inflammatory drugs, both non-selective and selective. The highest CVR was associated with diclofenac and indomethacin. Selective cyclooxygenase-2 inhibitors cause an imbalance between prostacyclin I2, which has vasodilatory and anti-aggregation properties, and thromboxane A2, a vasoconstricting substance, which stimulates thrombocyte aggregation, resulting in further increases in CVR [4].

### 3.6. The Role of RA as a Non-Traditional Cardiovascular Risk Factor

Taking into consideration the detailed concepts presented in previous sections, RA is gaining more and more recognition as a significant non-traditional cardiovascular risk factor. Not only does it directly affect the cardiovascular risk, but it can also modulate the effect other traditional factors have.

On one side, RA is highly associated with traditional CV factors, including tobacco use, hypertension, dyslipidemia, and diabetes mellitus. Additionally, smoking is an established key element in RA pathogenesis. Systemic inflammation alters the metabolic pathways involved in cardiovascular risk, creating favorable conditions for earlier atherosclerosis initiation and expeditious progression. In this case, RA, through its chronic inflammatory status, magnifies the effect of traditional CV risk factors, with subsequent augmentations in CV risk. The risk is further elevated by specific treatments used in this category of patients; for example, despite the fact that glucocorticoids, which are commonly used in RA therapy, lower the inflammation, they exacerbate the traditional risk factors.

On the other hand, the characteristic RA inflammation is an independent CV risk factor. Atherosclerosis, one of the most important contributors to the cardiovascular burden, is, in essence, an inflammatory process. This explains the relevance of RA in CVD development and progression, through a wide range of mechanisms, emphasized in all the pathophysiological stages of atherosclerosis: increased levels of pro-inflammatory cytokines, lipoprotein modifications, increased oxidative stress, leukocyte stimulation, and intensified endothelial dysfunction.

Similarly, the maladaptive inflammatory responses and immune dysregulation seen in other autoimmune disorders (including systemic lupus erythematosus, psoriatic arthritis, and inflammatory bowel disease) increase the cardiovascular risk through diverse micro-environmental alterations: autoimmune inflammatory reactions caused by immune complex deposits, persistent inflammation-induced oxidative stress, structural alterations due to chronic inflammation, and microvascular dysfunction. This complex interaction adds to the higher prevalence of traditional CV factors in these populations. The cardiovascular risk of autoimmune augmentation is particularly significant in younger women, who are predominantly affected by autoimmune disorders. Because this is a category of subjects generally less affected by atherosclerotic disease, the connection between autoimmune disorders and atherosclerotic cardiovascular disease has been a topic of great interest during recent years [42,43].

## 4. Frequent Cardiovascular Diseases in RA

Rheumatoid arthritis increases the risk of developing various cardiovascular diseases. The most frequent diseases include: acute coronary syndrome, in the form of myocardial infarction; arrhythmias, especially atrial fibrillation, and, consequently, ischemic stroke; and heart failure. The pathogenic associations between RA and these CVDs are detailed below.

### 4.1. Acute Coronary Syndrome—Myocardial Infarction

Atherosclerosis holds a pivotal role in the appearance of an acute coronary syndrome (ACS). Atherosclerosis is precipitated by altered lipid metabolism and enhanced oxidative processes, both stimulated by chronic RA inflammation. The incidence of clinical and even subclinical atherosclerosis is elevated in RA patients, with the risk of developing CVD being twice as high in RA patients compared to the general population. This specific population has a doubled risk of myocardial infarction (MI) and a 1.5-fold increased risk of cerebrovascular incidents [44].

Since inflammation accelerates the process of atherosclerosis, RA provides an increased risk of having an acute cardiac event, such as a myocardial infarction, even in the first years after the diagnosis. Similarly to the general population, the incidence of suffering from an MI increases with age and gender (male population). The increased incidence of MI tends to increase with RA duration. Holmqvist et al. [45] concluded RA determined an elevation of 40% in the risk of experiencing an acute coronary syndrome (HR = 1.41, 95% CI: 1.29–1.54). The risk remained significantly higher in RA patients even after stratification by gender and age. However, the risk was statistically increased in seropositive patients and those having at least a moderate-activity rheumatologic disease (DAS28 > 3.2). Even though authors observed a decline in the incidence of ACS over the years in RA patients, this was similar to the decline in incidence observed in the general population. Therefore, they concluded there was no significant decline in the excessive risk (attributed to RA inflammation) of MI in this specific population of patients. This decline could be associated with better control of the traditional cardiovascular risk factors (smoking, dyslipidemia, physical inactivity, and hypertension) in both populations. In this case, chronic inflammation (as seen in RA) seemed to be responsible for the excessive risk of ACS these patients have. Disease activity (especially at the onset of RA, before treatment initiation) holds a tremendous role in the onset of ACS, given the fact that inflammation lowers the stability of plaques and generates thrombus formation.

One study [46] compared the evolution of patients with RA suffering from an MI to patients without RA. Regarding the traditional risk factors, there were no significant differences between the two groups. In terms of severity, the MIs were also similar between patients with and without RA. Treatments (including reperfusion techniques and cardio-protective medication) were similar. The 5-year mortality rates were significantly greater in RA patients (57% ± 6%) compared to the non-RA group (36% ± 4%, *p* = 0.036). RA patients had a 50% chance of recurrent ischemia compared to the general population (HR = 1.51, 95% CI: 1.04–2.18). The difference in recurrent ischemia risk was more pronounced in RA patients with time after the first incident. However, the authors did not find any significant associations between various RA characteristics (disease duration, seropositivity, disease activity/severity, and RA-specific treatment) and long-term outcomes after an ACS.

Aspirin/Acetylsalicylic acid (ASA) is a non-steroidal anti-inflammatory drug that reduces inflammation and inhibits platelet aggregation. This drug is usually indicated in ischemic cardiomyopathy. Considering the endothelial dysfunction, accelerated atherosclerosis, and increased oxidation of LDL cholesterol, RA patients were thought to benefit from early ASA therapy, to potentially reduce the risk of ischemic events. A regression analysis revealed no significant association between ASA therapy and prevention of ischemic vascular events (myocardial infarction—*p* = 0.575; ischemic stroke—*p* = 0.291). Therefore, ASA does not protect against major ischemic cardiovascular events in RA patients [44].

To conclude, RA patients have a higher incidence of ACS due to inflammation-promoted atherosclerosis, with poorer long-term outcomes.

### 4.2. Atrial Fibrillation and Ischemic Stroke

While men with RA tend to be more affected by atherosclerotic coronary disease, women with RA have a higher incidence of arrhythmias, including atrial fibrillation [47].

Atrial fibrillation (AF) is the most common arrhythmia worldwide, resulting in doubled mortality and a 5-fold increased risk of ischemic stroke (IS). RA increases the risk of AF to 1.4-fold. RA patients have an elevated sympathetic activity, which stimulates atrial excitability and an autonomic imbalance. In this case, patients are predisposed to various arrhythmias, including atrial fibrillation. In RA, AF is a consequence of both electrical and structural remodeling (fibrosis) within atria; these alterations contribute to the development, maintenance, and recurrence of this abnormal rhythm. In addition, the abundance of pro-inflammatory cytokines modifies the electrophysiological properties of cardiac tissue, promoting a pro-arrhythmic substrate. A study found that for RA patients, it was more likely to have a rate-control approach to AF rather than a rhythm control (*p* < 0.001). This could be a possible explanation for this population’s increased risk of ischemic stroke [48,49].

Atrial fibrillation is a well-known risk factor for ischemic stroke. In the AF population, the risk of ischemic stroke is influenced by other CV and non-CV comorbidities (including RA). Oral anti-coagulants (OACs) can effectively decrease the risk of IS in the general population. However, OAC treatment seems to be underused in RA patients. In a study comparing two groups of patients with AF, with RA and without RA, stroke or transient ischemic attack (TIA) prior to AF diagnosis was significantly more prevalent in the RA population (*p* < 0.001). In addition, the CHA_2_DS_2_-VASc score (a score used to determine if OAC therapy is indicated in AF) was higher in the RA group. During the 3-month follow-up period (after AF diagnosis), patients with RA were less likely to receive OAC treatment, despite higher CHA_2_DS_2_-VASc scores (OR = 0.88, 95% CI: 0.80–0.97). This might be another possible explanation for the higher risk of ischemic stroke in RA patients with AF. Patients with both AF and RA had a higher risk of having an ischemic stroke (HR = 1.36, 95% CI: 1.13–1.62). The risk was similar after adjustment of various confounders (age, gender, hypertension, diabetes mellitus, atherosclerotic CVD, and OAC treatment). The risk was higher for men with RA compared to men not suffering from the rheumatic disease; however, in women, there was no significant difference in risk between the two groups (RA and non-RA) [48].

Even in the absence of AF, RA, like most rheumatic diseases, increases the risk of different types of stroke, especially in younger patients (<50 years) and seropositive patients. Cerebrovascular diseases are more common in RA patients compared to the general population. RA patients are at increased risk of stroke (OR = 1.64, 95% CI: 1.16–2.30, *p* = 0.005), especially for ischemic stroke (OR = 2.66, 95% CI: 1.24–5.70, *p* = 0.012). TNF-α is a key cytokine in the activation of endothelial cells, stimulating their pro-coagulant and pro-thrombotic functions, elevating the risk of ischemic stroke. A study evaluated factors associated with this heightened risk. The risk of stroke in RA patients increased with hypertension, myocardial infarction, and use of low-dose aspirin. RA-specific factors that determined elevations in the risk of stroke included total joint replacement (TJR; OR = 2.13, 95% CI: 1.14–3.95, *p* = 0.017) and HAQ score (OR = 2.04, 95% CI: 1.40–2.97, *p* < 0.001). While HAQ scores are influenced by other comorbidities, TJR remained significantly associated with IS risk after adjustment for various confounders (OR = 2.28, 95% CI: 1.13–4.58, *p* = 0.021). In terms of RA therapy, rofecoxib (OR = 2.32, 95% CI: 1.05–5.13, *p* = 0.037) and prednisone (OR = 2.03, 95% CI: 1.08–3.84, *p* = 0.029) increased the risk of IS. For prednisone use, the OR for stroke increases with daily dose. Both TJR and HAQ are measures of RA chronic inflammation cumulative damage [44,50].

In addition, RA patients have an increased rate of recurrent ischemic stroke compared to the general population (HR = 1.37, 95% CI: 1.12–1.67, *p* < 0.01). This is facilitated by increased levels of pro-inflammatory cytokines, such as TNF-α and IL-6. The risk is also elevated by a raised TG/HDL-c ratio, which was associated with RA. The risk is further increased if the patients are currently smoking (adjusted HR = 2.19, 95% CI: 1.33–3.62, *p* < 0.01). The risk increases with duration of smoking (>40 years), number of cigarettes/day (>20), and smoking pack-years (>30). The elevated TG/HDL-c ratio and smoking both accentuate atherosclerosis and endothelial dysfunction and increase the serum levels of pro-inflammatory cytokines (especially TNF-α), leading to an expanded recurrence risk of IS in the RA population [51].

### 4.3. Heart Failure

RA patients are at increased risk of heart failure (HF) due to chronic inflammation. This is particularly relevant because, as opposed to the well-functioning heart, the failing one secretes TNF-α, a pro-inflammatory cytokine, closing a vicious circle. In RA patients, factors contributing to HF are similar to those in the general population: age, gender (male), hypertension, tobacco use, diabetes, and myocardial infarction. In addition, there are specific RA factors that influence the decrease in cardiac function (disability index, pain). Studies confirmed the role of TNF-α in the pathophysiology of heart failure in RA: patients treated with anti-TNF agents less frequently developed HF (*p* = 0.03) [52].

Retrospective studies emphasized that patients with RA have twice as high an incidence of heart failure, an increased prevalence of HF, and, consequently, an increased mortality rate associated with HF. The most frequent causes of HF are hypertension and ischemic cardiac disease; both conditions have an elevated incidence in the RA population, as a result of inflammation-accelerated atherosclerosis. However, these factors might not be enough to explain the HF. Chronic inflammation stimulates a wide range of structural changes in cardiomyocytes, extra-cellular matrix, and microvasculature (hypertrophy, fibrosis, or endothelial dysfunction), with functional consequences (decreased cardiomyocyte contractility). Citrullinated proteins (anti-CCP antibodies) tend to accumulate within the interstitial tissue of the myocardium, with sequential fibrosis and compromised cardiomyocyte function. In addition, citrullination lowers the sensitivity of miofilaments to calcium, impairing myocardium contractility. Therefore, RA patients have a higher chance of developing HF, both ischemic and non-ischemic. Studies showed that seropositive patients have a 40% increased risk of HF (especially ischemic HF), in comparison to rheumatoid factor (RF)—negative patients. RF positivity is associated with more intense inflammation and higher disease activity. HF risk increases with RA duration: Mantel et al. [53] observed a rapidly increasing risk of non-ischemic HF in the first year after RA diagnosis, up to a 2-fold (HR = 2.06, 95% CI: 1.37–3.20), associated with higher erythrocyte sedimentation rate, disease activity score, and CRP (markers of inflammation). For ischemic HF, the risk increased considerably after 10 years of RA. Regarding corticosteroids, only the risk of non-ischemic HF was tripled by oral use [53,54].

Løgstrup et al. [54] confirmed the higher risk of HF in RA patients, specifically in the first year after diagnosis (HR = 2.28, 95% CI: 2.06–2.53); in the following years, the risk was lower, but still remained elevated by almost 40% compared to the general population (HR = 1.39, 95% CI: 1.30–1.49 within 1–5 years after RA diagnosis; HR = 1.38, 95% CI: 1.27–1.50 within 5–10 years after RA diagnosis). The authors observed that ischemic cardiac disease doubled the risk of heart failure in the RA population (HR = 2.06, 95% CI: 1.90–2.24). In the absence of ischemic cardiac disease, the authors found that the risk of (non-ischemic) HF in RA patients was increased by merely 23%.

In contrast to the general population, the diastolic function of the heart, rather than the systolic one, is more frequently altered in RA patients. In RA patients, heart failure tends to manifest more subtly, in comparison to the general population: patients experience less frequent dyspnea on exertion, orthopnea, paroxysmal nocturnal dyspnea, and hepato-jugular reflux. Instead, they present more commonly with rales on auscultation. These differences in signs and symptoms could be attributed to RA (patients are less likely to exercise; rheumatic lung disease). The most important difference refers to a higher number of patients with heart failure with preserved ejection fraction (HFpEF), an indicator of diastolic dysfunction (impaired ventricular relaxation and filling). However, despite the preserved ejection fraction, RA-HF patients have a doubled one-year mortality rate compared to non-RA-HF patients [55].

### 4.4. Cardiac Valvular Disease—Aortic Valve

Valvulopathies have a higher incidence in the RA population, with almost one-third of these patients suffering from valvular heart disease [56].

Heart valves encounter a wide range of modifications due to the chronic inflammatory status of RA; the most common structural and functional changes include valve nodules, valve thickening, valve regurgitation, and stenosis. The most frequently involved valves are the mitral valve (predominantly mitral regurgitation) and the aortic valve (aortic stenosis and/or regurgitation) [57,58].

RA doubles the risk of having valvular regurgitation: mitral regurgitation—HR = 1.98 (95% CI: 1.69–2.31) and aortic regurgitation—HR = 2.12 (95% CI: 1.68–2.67). Rheumatoid arthritis generates leaflet fibrosis and nodules, sometimes extended into the valve rings and the subvalvular apparatus, causing valvular regurgitation. These are the consequences of CD4+ T lymphocytes and macrophages infiltration within valvular tissue. Release of TNF-α by macrophages triggers inflammatory processes within the valve, activation of valve interstitial cells, and calcium accumulation, impairing proper valve closure [57,58].

An RA-specific increasing risk of aortic stenosis (AS), specifically degenerative AS, has gained more recognition in recent years. One study [59] found that patients with RA had a significantly higher incidence of developing degenerative aortic stenosis (*p* < 0.05), with a 54% increase in risk (HR = 1.54, 95% CI: 1.28–1.85). The risk increased with rheumatic disease duration.

Degenerative aortic stenosis is the result of inflammation, lipid infiltration, and fibro-calcification. Pro-inflammatory conditions, including RA, stimulate the coagulation processes, leading to fibrin accumulation within valve tissue, with progressive thickening of the leaflets, reduced motility, and narrowed valve orifice [60].

Degenerative AS etiology is widely similar to that of atherosclerosis. Important risk factors include age, tobacco use, increased BMI, hypertension, dyslipidemia, and diabetes; all of these are traditional cardiovascular risk factors, which are more prevalent in the RA population [60].

Inflammation holds a significant role in the pathogenesis of degenerative aortic stenosis. The elevated serum levels of pro-inflammatory cytokines (including TNF-α, IL-1β, and IL-6) stimulate the osteogenic differentiation of valvular tissue, leading to remodeling and consequent development and progression of aortic stenosis. AS risk associations have been found with heightened ESR and CRP (HR = 1.11, 95% CI: 1.01–1.22)—as biomarkers of inflammation—and with therapy with biologic/targeted synthetic DMARDs (HR = 1.22, 95% CI: 1.07–1.39) or glucocorticoids (HR = 1.19, 95% CI: 1.08–1.32). These findings support the hypothesis that the more aggressive the rheumatic disease is (suggesting a greater degree of inflammation), the higher the risk of AS is, alongside less favorable evolution and prognosis [59,61].

Another possible RA-related factor that can elevate the risk of degenerative AS is anti-CCP positivity. One study [62] investigated the possible association between anti-CCP positivity and the risk of degenerative AS. Patients with degenerative AS were much more likely to be seropositive for anti-CCP (*p* = 0.035). Seropositive patients were divided into two groups: low-titers (with anti-CCP levels above the normal limit, but below the median value) and high-titers (with anti-CCP levels equal to or more than the median value). AS progression was more frequent in the high-titer group than in the low-titer or negative groups (19% vs. 11.3% vs. 8.4%, *p* = 0.041). Therefore, high positivity for anti-CCP was a relevant factor in AS evolution, with a more than doubled risk of AS progression (OR = 2.312, 95% CI: 1.006–5.310, *p* = 0.048). However, differences in all-cause mortality, cardiovascular death, or aortic valve replacement rate were not found between the two groups. Citrullination is an inflammation-dependent process that is widely associated with rheumatoid arthritis. Biopsies of affected aortic valves emphasized the presence of citrullinated proteins within the degenerated connective tissue of the valve, in accordance with high serum levels of anti-CCP antibodies. Therefore, anti-CCP antibodies could be a new biomarker for predicting AS’s possible development and progression [62].

RA patients also have a heightened risk of AS-related interventions and AS-related deaths. RA patients have a 34% increased chance of having an AS-related replacement intervention (HR = 1.34, 95% CI: 1.22–1.48), including SAVR (surgical aortic valve replacement) and TAVR (transcatheter aortic valve replacement). They also have a higher risk of AS-related death (HR = 1.26, 95% CI: 1.04–1.54). This underscores a more accelerated evolution of the valvular disease, requiring procedural treatment earlier, in comparison to non-RA patients [61].

### 4.5. Peripheral Artery Disease

Peripheral artery disease (PAD) is another consequence of atherosclerosis, being seen as an equivalent to coronary artery disease. PAD usually affects the medium- and large-sized blood vessels, most frequently in the lower extremities. The easiest method to establish the diagnosis is the ankle-brachial systolic pressure index, with a threshold value of ≤0.9 for PAD [63,64].

Similarly to other atherosclerotic diseases, PAD further increases the mortality rate of the RA population, with a two to six-fold elevation in the risk of cardiovascular and cerebrovascular events [64].

As mentioned in previous sections, atherosclerosis, a systemic process affecting blood vessels throughout the body, is triggered and maintained by inflammation. Therefore, both the initiation and progression of PAD are determined by inflammation. The inflammatory pathways are stimulated by elevated levels of oxidative stress and enhanced by traditional risk factors, such as hypertension, smoking, dyslipidemia, and diabetes mellitus (which are more prevalent in RA patients) [65].

Endothelium dysfunction is the first step in the atherosclerotic process. Endothelial damage is augmented in RA patients due to chronic inflammatory aggression on endothelial cells. Traditional CV risk factors (hypertension, tobacco use, dyslipidemia, diabetes) contribute to the development and progression of the peripheral artery disease. RA patients have higher systolic and diastolic blood pressures, increasing the sheer force on the endothelium. Abnormalities in lipid metabolism contribute to endothelial dysfunction and to arterial stiffness. Endothelial dysfunction is the consequence of an imbalance between injury processes and its regenerative capacity. Long-standing exposure to oxidative stress, as seen in chronic inflammatory diseases, alters the ability of the endothelium to regenerate. Chronic inflammation magnifies the effect of traditional CV risk factors, leading to accelerated atherosclerosis. The chemical alterations seen in RA, with increased levels of pro-inflammatory cytokines, make the endothelium more prone to vasoconstriction, cell proliferation, and thrombosis. The complex interaction between these pathophysiological processes determines plaque formation in the peripheral arterial bed [66].

RA patients have a 73% higher chance of developing PAD (HR = 1.73, 95% CI: 1.57–1.91). The risk is even higher for younger patients (<50 years)—HR = 3.23 (95% CI: 2.55–4.09). Associations with other comorbidities/traditional CV risk factors augment the risk even more: diabetes mellitus (HR = 4.04) and hypertension (HR = 2.55)—which are well-known independent risk factors for PAD. The more comorbidities RA patients have, the higher the risk of PAD is. The highest risk of PAD in RA patients is in the first year after diagnosis (HR = 2.42, 95% CI: 1.92–3.05); even though there is a decline in risk in the following years, it still remains heightened, in comparison to the general population (HR = 1.45, 95% CI: 1.17–1.81). These results underscore the importance of RA inflammation in the pathophysiology of PAD [63].

### 4.6. Vasculitis

As mentioned before, the chronic and systemic inflammation in RA affects multiple organs throughout the body, including the blood vessels, specifically their wall structure, leading to rheumatoid vasculitis (RV). Small blood vessels are commonly affected, but this severe condition can also appear in medium- or large-sized vessels. It usually appears in patients with severe, long-standing (more than a decade, in general), seropositive and uncontrolled RA. It is associated with higher morbidity and mortality rates. Almost half of the patients with RV die within 5 years of diagnosis due to complications of the disease and/or the required immunosuppressive medication [67,68].

The clinical features of RV are extremely diverse, considering it can affect vascular beds in any organ. The most frequently involved organs are the skin, eye, gastrointestinal tract, lung, kidney, and central and peripheral nervous systems. When skin is affected (almost in all patients with RV), individuals experience non-healing leg ulcers, palpable purpura, isolated nail infarcts, and digital ischemic lesions. Vasculitis of vasa nervorum (with necrosis and occlusion) in the peripheral nervous system leads to distal sensory and/or motor neuropathy [67].

RV involves inflammation of the vessel wall, subsequent injuries, necrosis, thrombosis, and tissue ischemia. Its pathogenesis involves the presence of immune complexes within the wall structure and activation of the complement system. These pathways are supported by the typical appearance of RV in RF-positive patients, with low serum complement levels [67].

Biochemically, RV is associated with an increased inflammatory response, emphasized by elevated plasma levels of inflammation biomarkers (CRP and ESR). Patients also present with high levels of circulating auto-antibodies (RF in particular), which facilitates the formation and deposition of immune complexes within vessel walls. This is an essential pathway involved in RV appearance and progression; it triggers further inflammatory processes, with more complex deposition, closing a vicious pathophysiologic circle [68].

In addition, associations between anti-CCP antibodies and rheumatoid vasculitis have been found. ACCP high titers are significantly more prevalent in RA patients with vasculitis manifestations, in comparison to RA patients without vasculitis (*p* = 0.03). Titers over 15 IU/mL were associated with both high sensitivity (85.2%) and specificity (98%) in RV diagnosis, supporting the potential role these antibodies could have as a differential diagnosis biomarker of systemic vasculitis [69].

Studies showed there are a few risk factors contributing to RV development. These factors include a younger age at the diagnosis of the rheumatic disease, currently smoking at the RA diagnosis, affections of the peripheral vessels, cerebrovascular disease, severe forms of RA (including nodules, erosions, and joint surgeries), and biologic therapy. Younger age at diagnosis implies more exposure to inflammation. Vascular comorbidities, such as cerebrovascular and peripheral artery disease, can increase the risk of RV by four to six times, by adding to the vascular burden [68].

Smoking can explain the higher chance of developing RV through its implications in inflammation and oxidative stress processes, accentuating the vascular structural damage. Smoking can double the risk of developing RV (OR = 2.04, 95% CI: 1.04–3.97) [68,70].

Biologic therapy can be indirectly associated with RV risk. In general, this treatment option is indicated in severe cases, unresponsive to first-line therapy (glucocorticoids and conventional synthetic DMARDs, including methotrexate and hydroxychloroquine) [68].

Genetic factors also seem to have an impact on the risk of RV. Studies [70] showed significant associations of RV with higher frequencies of HLA-C3 alleles (*p* < 0.001), and lower frequencies of HLA-C7 alleles (*p* = 0.018). The chance of having RV in patients with the HLA-C3 genotype is four times higher (OR = 4.15, 95% CI: 2.14–8.08). While other studies [71] underscored the connection between HLA-DRB1*04 (a very well-known genetic risk factor for RA, especially severe forms) with extra-articular manifestations (including RV—OR = 2.44, 95% CI: 1.22–4.89), this study emphasized that HLA-C3 significantly increase the risk of vasculitis in patients with HLA-DRB1*04 shared epitope genotype (OR = 2.7, 95% CI: 1.2–6.1), and in patients without the HLA-DRB1*04 shared epitope genotype (OR = 8.3, 95% CI: 2.0–34.2). The association between HLA-C3 and vasculitis emphasizes the importance of class I MHC (major histocompatibility complex) genes in the pathophysiology of this extra-articular manifestation. HLA-C3 is involved in T cell activation, stimulating chronic inflammation and its subsequent vascular damage.

Protective factors against RV can be hydroxychloroquine treatment (OR = 0.54, *p* = 0.03), possibly due to its anti-inflammatory and immunomodulatory properties, and low-dose aspirin (OR = 0.42, *p* = 0.02), as a result of its anti-platelet aggregation and thromboxane A2 inhibition [68].

In light of this evidence, despite a lower incidence in recent years, rheumatoid vasculitis still remains an important vascular complication of RA, making patients susceptible to various complications, increasing mortality, and decreasing quality of life. These features highlight the significance of RA disease proper control, RV early diagnosis, and adequate treatment.

In conclusion, not only do RA patients have a higher incidence of various cardiovascular diseases, but they also have a much higher cardiovascular mortality rate in comparison to the general population. These phenomena are probably due to the chronic inflammatory state of this rheumatic disease.

## 5. Coronary Microvascular Disease

In rheumatoid arthritis, constant inflammation is a determinant of coronary microvascular disease, defined by low myocardial flow reserve. This parameter is assessed by cardiac positron emission tomography and is associated with an increased risk of major cardiovascular events (including cardiac death) [72].

Weber et al. [72] assessed in their study how anti-inflammatory therapy influences coronary flow reserve using data from the LiiRA trial. A cohort of patients with RA was followed for the assessment of myocardial flow reserve (MFR), at the same time that the anti-inflammatory treatment with TNF-α inhibitors (certolizumab) was initiated. The coronary reserve (expressed as the ratio of peak blood flow in the coronary arteries during stress to resting blood flow) is considered mildly reduced at values below 2.5, moderately reduced at values below 2, and severely reduced at values of the ratio below 1.5. The MFR level was recorded at baseline (reference level), both at rest and during maximal hyperemia (obtained with regadenoson). The second MFR assessment took place 24 weeks after the start of the biologic treatment. Coronary microvascular disease was defined as MFR < 2.5. Compared to the control group, RA patients had an 18% lower MFR level (*p* = 0.011). These values indicate mild microvascular damage. Following treatment with TNF-α inhibitors, there was no significant difference in MFR compared to pre-treatment levels. Only acute-phase reactants (ESR and high-sensitive CRP) and the levels of IL-6, IL-1b, and soluble TNF type 2 receptors showed significant decreases following the biological treatment (*p* < 0.05). In the case of RA patients who met criteria for microvascular disease initially (MFR < 2.5), there was some correlation between reduced IL-1b levels and increased myocardial flow reserve (Spearman coefficient = −0.33, *p* = 0.067). Following these results, the authors found that treatment with TNF inhibitors leads to a reduction in inflammatory markers, but it does not significantly influence coronary microvascular disease. The authors emphasize the need for subjects with higher CVR (more severe microvascular dysfunction) to objectify a statistically significant improvement in MFR after the biologic treatment. However, in a subgroup of patients, they observed improvements in subclinical myocardial damage and possibly in microvascular disease by regulating the pathway of IL-1b [72,73].

Liao [74] and collaborators assessed the prevalence of coronary microvascular disease (identified as MFR < 2) in patients with RA and its association with increased cardiovascular risk. Over half of the patients with RA were diagnosed with coronary microvascular disease, with no significant difference between the two genders. A statistically significantly higher mortality rate was observed in patients with microvascular damage (*p* < 0.0001). The presence of microvascular disease was associated with an increased risk of all-cause mortality (HR = 2.4, 95% CI: 1.4–4.2) after adjusting for other cardiovascular risk factors. In rheumatoid arthritis, the damage to coronary microvascularization is caused by the persistent inflammatory status, which is accompanied by changes in the endothelial function (as evidenced by parameters such as flow-mediated vasodilation). This hypothesis is also supported by the treatment with anakinra, an IL-1 antagonist, which reduces inflammation and improves vasodilation and coronary flow reserve. Persistent/recurrent episodes of exacerbation lead, over time, to endothelial dysfunction and subclinical myocardial dysfunction, which predisposes to an increased risk of myocardial infarction. Thus, the myocardial coronary reserve becomes an important quantitative marker of cardiovascular risk, with prognostic function. Reducing this parameter puts RA patients at increased risk of MACE (major adverse cardiac events), including myocardial infarction and death.

## 6. Cardiovascular Risk Assessment in Patients with RA

Regular cardiovascular assessment of patients with rheumatoid arthritis should be preeminent in the monitoring of this category of patients. The physical examination should include the assessment of the heart and blood vessels, and blood pressure measurements. The biologic assessment should include the lipid profile and inflammatory markers. A carotid ultrasound should be performed for the early identification of atherosclerosis (by increased intima–media thickness). Bedeković et al. [2], following a comparison between the number of cardiovascular deaths in the rheumatoid arthritis population and the general population, emphasized the underestimation of cardiovascular risk by the classical CVR estimation scores. The main cause of death was chronic heart failure, with acute pathology playing a less important role in the mortality of this category of patients [1].

The difficulty of accurately estimating cardiovascular risk assessment in patients with RA is the result of several conditions. Patients with an elevated inflammatory status are at the highest risk, despite the fact that inflammation lowers the serum cholesterol levels. Rheumatoid cachexia boosts the cardiovascular risk more than obesity, and the possible excess of adipose tissue may also be represented in a form other than abdominal obesity. These sources of error limit the use of risk scores derived from the general population in patients with rheumatoid arthritis [75].

When assessing the CVR in the general population, the European Society of Cardiology (ESC) suggests taking into consideration five aspects: the severity of potential arterial hypertension, the presence of a metabolic disease (DM), additional CVR factors, end-organ failure, and cardiovascular complications. The presence of a possible chronic inflammatory disease or its treatment (with glucocorticoids and/or NSAIDs) is not included in the general categories, leading to improper CVR stratification [76].

### 6.1. Cardiovascular Risk Assessment Using Standard Matrices

The assessment of cardiovascular risk in patients with rheumatoid arthritis using standard matrices (Framingham risk score and SCORE model) tends to undervalue this risk in patients in the low/intermediate risk groups, while overestimating the risk in those at high risk. As rheumatologic impairment is in itself a cardiovascular risk factor, recent studies and ESC recommend multiplying the relative risk (calculated with standard matrices) by 1.4 for men and 1.5 for women, respectively, when at least two of the following criteria are met: (1) duration of rheumatologic disease > 10 years; (2) RF/ACCP seropositivity; (3) presence of extra-articular manifestations (Felty’s syndrome, pericarditis, pleuritis, polyneuropathy, mononeuritis, episcleritis, glomerulonephritis, and vasculitis) [2,77].

The Framingham risk score (FRS) also tends to underestimate the cardiovascular risk in patients with RA, especially in women (by 102%; in men by 65%). Thus, the real cardiovascular risk is significantly higher than predicted. This major difference was more common in older patients, those with positive RF, and those with persistently elevated ESR values. In addition, in the case of patients with RA, the Framingham risk score is not able to adequately differentiate between low- and intermediate-risk patients. The compelling difference between the predicted and observed cardiovascular risk score may be due to the different actions of traditional risk factors in RA patients. For example, despite the high incidence of smoking in rheumatologic patients, it appears to have less of an effect on cardiovascular diseases. A similar effect occurs in the case of dyslipidemia, with the lipid paradox described above [78].

The Reynolds risk score (RRS) includes, in addition to the traditional CV risk factors (age, gender, lipid profile, blood pressure, smoking status), variables related to inflammation (high-sensitive CRP) and genetic risk (family history of myocardial infarction before the age of 60 years). The former variable, significant in regard to RA patients, was able to reclassify healthy women from the “intermediate risk” group to either the low or the high risk group. This facilitates a more optimal treatment strategy, with maximal benefits and minimal toxicity. This risk model was studied for application in male individuals from the general population. It performed better at CV risk assignment to groups, in comparison to traditional risk scores (which do not include markers of inflammation). Almost one-fifth of the patients were reassigned from intermediate risk to lower or higher categories, with impressive accuracy (>80%) in reclassification. Other studies show that the Reynolds risk score tends to underestimate the cardiovascular risk in women with RA, despite the fact that it includes the value of the C-reactive protein [78,79].

A study [80] following a Dutch cohort of RA patients compared CVR assessment, using the most common risk scores: FRS, SCORE, RRS, and QRISK2. The FRS is more commonly used in American cohorts, whereas SCORE is particularly used in European countries. Both algorithms include traditional risk factors and estimate the 10-year risk of CVD. All four algorithms encountered differences between the observed and the predicted CVR. Despite the excellent negative predictive value, ranging between 92 and 97% (depending on the model and the cut-off value), up to 24% (SCORE) and 32% (RRS) patients assigned to the low-risk group (<10%) still experienced a CV event. Authors concluded that, while SCORE, FRS, and RRS tend to underestimate the risk, QRISK2, which includes RA as a risk factor, overestimates the probability of a future cardiac event. The underperformance was most important in the two-thirds with the lowest estimated CVR. Unfortunately, these are the most important groups for risk stratification, with regard to specific treatment initiation. Areas under the ROC curve were around 0.78–0.80, indicating a moderate capacity of discrimination. The RRS, in spite of including CRP serum levels as a risk factor, did not perform much better in CVR estimation. Interestingly, the FRS, a risk score developed and tested on the American population, had the best performance on the European population studied by Arts et al. [80], with the lowest percentage of “low-risk” misclassified patients. In order to improve the accuracy of risk discrimination, several approaches have been proposed. One of them suggested altering the cut-off points in CVR to which treatment strategies start to be considered/recommended. While this approach could improve the primary cardiovascular prevention, polypharmacy and substance interactions should be kept in mind (as RA patients are already on treatment for the rheumatic disease).

Various approaches have been suggested to improve the accuracy of cardiovascular risk scores in patients with RA: from multiplying scores obtained with standard matrices, including certain biomarkers, to modifying the impact of certain parameters in risk calculation [81].

Some studies have assessed whether multiplying the standard score by a given constant gives a more accurate estimate of the risk. While this results in better calibration (a smaller difference between predicted and observed risk), it does not provide a better classification in a cardiovascular risk category. Other researchers have modified the standard risk scoring (SCORE) matrices, giving each parameter a different weight in the risk calculation; this approach also did not enhance the discriminatory potential and, in addition, resulted in an underestimation of the cardiovascular risk [81].

Chronic inflammation might alter the impact of traditional cardiovascular disease factors on risk evaluation. Arts et al. [82] studied patients with RA from a Dutch cohort. They compared the estimated cardiovascular risk obtained by the original SCORE, a recalibrated SCORE, and a modified SCORE. The recalibrated SCORE took into consideration only the traditional CV risk factors, with different weights in risk estimation. The adapted SCORE included several specific variables (BMI, hypertension at baseline, DM at baseline, a high DAS28 score > 5.1 at baseline), alongside traditional risk factors (current smoking status, systolic blood pressure, TC:HDL-c ratio). Median 10-year CV risk with original SCORE was 9.1% (2.7–26.6%), while the adapted SCORE delivered a risk score of 6.7% (1.6–18.4%). Discrimination capacity was similar between the three scores (for original SCORE AUC = 0.78, 95% CI: 0.74–0.82; for recalibrated SCORE AUC = 0.78, 95% CI: 0.74–0.83; for adapted SCORE, AUC = 0.80, 95% CI: 0.75–0.84). The original SCORE tended to underestimate risk of patients in the low and intermediate risk categories, while overestimating the risk of those in the high risk group. The recalibrated algorithm underperformed in all categories, while the adapted matrix performed similarly to the original one. Even though the adapted SCORE managed to reclassify some patients in their appropriate risk group, the majority of subjects (more than two-thirds) were not reclassified into a different category of risk. In light of these results, neither the recalibration nor the adaptation of the original SCORE led to significant improvement in CVR estimation in RA patients.

A possible cause for underestimation of the CVR in RA patients is the higher baseline risk in this population (compared to subjects of the same age and gender). The chronic inflammation already creates a risk of CVD development, to which traditional CV risk factors and other RA-specific factors are added [82].

The inclusion of specific biomarkers also did not lead to notable improvements in the discrimination of the Framingham risk score, according to a study carried out on a Swedish cohort of RA patients [81]. The inclusion of autoantibodies and inflammatory markers (CRP, RF, ACCP, oxidized LDL, NTproBNP) had modest results as far as the prediction of cardiovascular risk was concerned. Only the inclusion of anti-apolipoprotein A-I antibodies (anti-ApoA-I) resulted in a significant (175%) improvement in cardiovascular risk discrimination (AUC increased from 0.72 for the simple FRS to 0.81). This laboratory parameter is easily measurable, which supports its potential in correctly identifying RA patients at high cardiovascular risk. Both anti-apoA-I and NTproBNP antibodies indicate the presence of vulnerable atherosclerotic plaques and ischemic heart disease [81].

RA-specific risk scores have also been developed: ERS-RA, which includes parameters specific to rheumatologic pathology—disease activity (CDAI), disability (modified HAQ), daily prednisone use, and disease duration (>10 years). This evaluation matrix showed a significantly higher discerning ability than the standard scores (increase in the C-index statistic from 0.73 in the basic model to 0.76 in the extended model). This score allows for a better classification of patients in the cardiovascular risk categories, which facilitates appropriate therapeutic management. The accuracy of this score is augmented when there is information on the patient’s smoking status. Overall, this score allowed for a correct identification of patients at a high enough risk to require intervention on the cardiovascular risk factors (CVR ≥ 7.5%). However, performance drops when it comes to subjects whose cardiovascular risk exceeds the threshold of primary prevention [30,74,81].

Another study [83] compared the performance of the ERS-RA score and the American College of Cardiology/American Heart Association (ACC/AHA) score in a cohort of homogeneous patients, in terms of rheumatic disease duration. The ERS-RA calculator includes both traditional risk factors (age, gender, smoking, dyslipidemia, diabetes, and hypertension) and RA-specific factors (Clinical Disease Activity Index > 10, modified HAQ > 0.5, corticoid use, rheumatic disease duration > 10 years). The ERS-RA was calculated in two sets—the first one included hypertension and dyslipidemia reported by either the physician or the patient, while the second set included measurements of blood pressure and lipids, alongside reported diagnosis and/or specific treatment. The ACC/AHA score, used for comparison, is based on variables such as age, gender, antihypertensive treatment, blood pressure, total cholesterol and HDL-cholesterol, diabetes, tobacco use, and race. Authors compared the ERS-RA scores with both the original ACC/AHA scores and the modified ACC/AHA scores (i.e., ACC/AHA × 1.5) [83,84].

Both variants of ERS-RA (reported and measured) and both variants of ACC/AHA (original and modified) revealed similar and moderate discriminatory capacity, with an AUC of 0.77 (95% CI: 0.72–0.82) for ERS-RA reported, 0.76 (95% CI: 0.71–0.81) for ERS-RA measured and 0.77 (95% CI: 0.72–0.83) for both variants of ACC/AHA. For patients with a risk of 5–15%, discrimination was less than optimal, with an AUC ranging from 0.52 (0.43–0.61) for the reported ERS-RA to 0.59–0.60 for the measure ERS-RA and the ACC/AHA × 1.5 scores. Unfortunately, patients with this CV risk are the ones for whom accurate estimation is of the utmost importance, leading to a proper primary prevention strategy [83].

Both ACC/AHA and the measured ERS-RA were able to reclassify patients incorrectly assigned to the low-risk group by the reported ERS-RA. The measured ERS-RA had the highest positive and negative predictive values; however, compared to the other risk scores, the differences were indeterminable. In high-inflammatory patients (defined as a persistent ESR ≥ 40 mm/h), all four scores performed poorly in discriminating patients based on their cardiovascular risk. A low performance in this subset of patients is to be expected from the ACC/AHA scores, which are based only on traditional risk factors; the ERS-RA scores include, otherwise, parameters of the inflammatory status, but they did not outperform the standard matrices. This could be the result of the dichotomous quality of the inflammation markers (CDAI > 10, modified HAQ > 0.5) [83].

The results of this study underlined the non-superiority of the ERS-RA score in CV risk stratification, in comparison to the ACC/AHA scores. The least error of underestimation was registered for the modified ACC/AHA score, corresponding to the EULAR recommendations (multiplication by 1.5 of the ACC/AHA score). The weakest performance belongs to the reported ERS-RA matrix, with nearly a quarter of CV events classified as low risk by it [83].

Another possibility for enhancing the accuracy of CV risk estimation is represented by including the coronary artery calcium score (CAC). In the general population, CAC is equivalent to subclinical atherosclerosis and is an independent predictor of future atherosclerotic CVD. RA patients have elevated CAC. Despite this, they are usually attributed to the low-risk category, being deprived of prevention therapy (with statins). Measurement of CAC can be used in cases of uncertainty regarding accurate risk stratification. High CAC (≥300 Agatston units or ≥75th percentile for age, gender, and ethnicity) is consistent with high CV risk [85].

One study followed RA patients with high CAC, comparing risk stratification through FRS, RRS, and the ACC/AHA 10-year risk score. The ACC/AHA risk score intends to classify patients aged 40–75 years, without DM or atherosclerotic CVD, with LDL-cholesterol levels < 190 mg/dL into risk groups. A 10-year risk ≥ 7.5% using this model recommends initiation of lipid-lowering treatment. The authors considered subjects to be at high CV risk if their risk scores exceeded 10% (for FRS and RRS) and 7.5% (for ACC/AHA). A total of 35% of the patients eligible for ACC/AHA risk categorization had high CAC. The CAC was associated with more traditional risk factors (age, male gender, and dyslipidemia) and with specific markers of inflammation (high CRP levels). In terms of accurate assignment of “high CAC” patients to the elevated risk group, the ACC/AHA scoring performed better: 41% of the subjects were included in this group; on the other hand, both FRS and RRS managed to include only a third of the “high CAC” patients in the high-risk group. But the ACC/AHA score included 28% of the “non-high CAC” patients in the high-risk group. The predictive capacity of a high CAC was similar between the three scores. Even though the ACC/AHA was able to reclassify 13% of the patients into a different risk group (first assigned by FRS/RRS scoring), there was no statistically significant difference in the ability of the three scores to allocate RA patients to the appropriate risk category. The authors investigated the possibility of reducing the threshold for elevated CV risk from 7.5% to 5%, with regard to more accurate risk stratification in RA patients (considering there was a baseline CV risk induced by chronic inflammation). This alteration did manage to reallocate more patients with high CAC into the increased risk group (53%); however, it did reallocate more than one-third (38%) of the patients without high CAC into the same risk group [84,85].

The ACC/AHA scores facilitated the identification of high-risk RA patients, who need lipid-lowering therapy by almost 10%, in comparison to the Framingham score (41% instead of 32%). While the difference did not reach statistical significance, it was still relevant from a clinical point of view. But, in spite of its lower threshold for elevated CV risk (7.5% compared to 10% for the FRS and RRS), the ACC/AHA score still failed to classify almost two-thirds of subjects with high CAC into the high-risk group [85].

Since the presence of a high CAC is equivalent to an increased cardiovascular risk, this imaging biomarker might prove to be useful in risk stratification in particular categories of subjects (including RA), considering the low performance of standardized matrices (applicable in the general population). This analysis could be of help until better markers are identified [85].

Taking into consideration the information presented above, in spite of a wide range of CVR assessment scores, it has not yet been identified which is best to use. In this case, cardiologists and rheumatologists should follow the recommendations of the European Alliance of Associations for Rheumatology (EULAR) when evaluating the cardiovascular risk of an RA patient.

### 6.2. EULAR Recommendations

**Figure 2:**
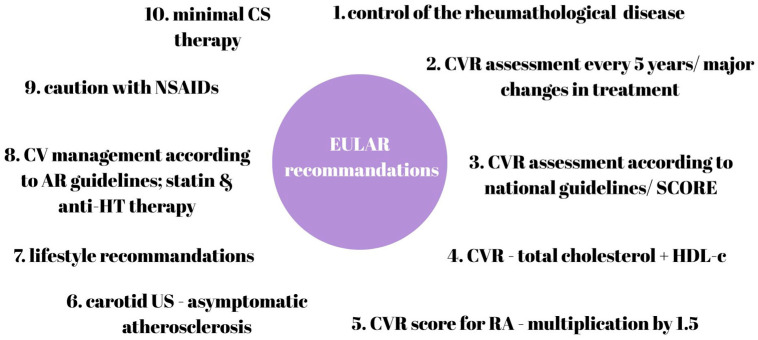
EULAR recommendations: 1. Reducing inflammation lowers the cardiovascular risk. The recommended treatment to reduce inflammation consists of conventional synthetic (in particular, methotrexate) and biologic (such as TNF-α inhibitors) DMARDs. Rituximab and tocilizumab lead to a reduction in carotid intima–media thickness, a marker of CVR. 4. Lipids should be measured when the rheumatologic disease is stable/in remission: the relationship between serum lipids and cardiovascular risk is not linear in this category of patients. 5. Adapting the cardiovascular risk scores for RA patients by multiplying them by 1.5, if the rheumatologic disease is not already included in the algorithm as a risk factor (e.g., QRISK2, QRISK3). There are no specific criteria to be met for the application of this multiplication factor. 6. Carotid atherosclerotic plaques are associated with future acute cardiac events. Plaque size and vulnerability vary in direct proportion to the duration and activity of the rheumatologic disease. 7. Lifestyle recommendations: a healthy, balanced diet (e.g., Mediterranean diet), regular physical activity, and smoking cessation. Physical activity reduces inflammation and the levels of serum C-reactive protein. It is also accompanied by improvements in micro- and macrovascular functions. 8. Certain drugs used in the treatment of RA (NSAIDs, corticosteroids, cyclosporine, and leflunomide) lead to high blood pressure. Statins reduce serum cholesterol levels, atherosclerotic burden, and cardiovascular morbidity and mortality; they have anti-inflammatory effects beneficial to RA patients. 10. Cardiovascular risk increases with higher daily dose of corticosteroids, increased cumulative dose, and longer exposure to corticosteroid therapy ([86] modified).

The European Alliance of Associations for Rheumatology created a list of recommendations for the management of cardiovascular risk in patients with RA, which are presented in Figure 2 [86].

## 7. Cardiac Imaging in Cardiovascular Risk Stratification

Patients with RA have an increased prevalence of subclinical atherosclerosis. This can be evidenced by assessing the presence of atherosclerotic plaques in the carotid arteries, which is equivalent to coronary artery disease. Appropriate stratification of RA patients according to cardiovascular risk is hindered by the presence of asymptomatic atherosclerosis (manifested by the presence of carotid atherosclerotic plaques). Using the mSCORE model (SCORE risk multiplied by 1.5) is not sufficient to adequately characterize cardiovascular risk in RA patients. The presence of carotid plaques can be used as a surrogate for increased cardiovascular risk, as recommended by the ESC (class I recommendation, evidence level A) [87].

Plaques in the common carotid artery, internal carotid artery, or carotid bulb were defined as intraluminal protrusions ≥1.5 mm (longitudinal section) when both walls had sharp edges or when the protrusion was at least double the adjacent intima–media thickness. Patients included in Semb’s [87] study were asymptomatic, with insignificant carotid stenosis (<50%). The cardiovascular risk of patients with RA was calculated according to the EULAR recommendations: multiplication of the SCORE model score by 1.5 (modified SCORE risk). When the SCORE risk was used to classify patients into risk categories, lipid-lowering management was performed according to Table 1.

Researchers observed that when the presence of carotid atherosclerotic plaques was also taken into consideration, more than one third of patients with a cardiovascular risk that was considered low/moderate initially (with no indication for lipid-lowering treatment) were redistributed into the category of patients requiring intensive treatment with statins. In addition, more than 50% of patients belonging to the “considered treatment” group were reassigned to the “recommended treatment” group after including carotid plaques in the risk assessment [87].

Statin treatment reduces the risk of cardiovascular events by approximately 30%. When LDL-cholesterol targets are reached, the volume of the carotid plaques also decreases. Thus, the inappropriate classification of RA patients as “no treatment”/”low/moderate potency treatment” leads to the progression of atherosclerotic disease and consequent increase in cardiovascular risk [87].

Subclinical atherosclerosis is represented by increased intima–media thickness in the carotid artery. The increase in this parameter is associated with increased inflammatory stress. There is a significant tendency for carotid atherosclerotic plaques to grow in size with the increase in ESR and C-reactive protein values. The presence of atherosclerosis at this level has been associated with the duration of the rheumatologic disease, the age of the patients, and elevated systolic blood pressure. High-risk atherosclerotic plaques show calcifications, a necrotic lipid core, neovascularization, and inflammatory cell infiltrate. Plaque progression and destabilization are associated with high serum levels of pro-inflammatory cytokines (IL-6 and TNF-α) [88,89,90].

In conclusion, carotid ultrasound assessment ensures a better classification of RA patients, especially women, in the cardiovascular risk categories. The main consequence of this non-invasive examination is to ensure an appropriate lipid-lowering treatment, which leads to the regression of the atherosclerotic disease and the reduction in the cardiovascular risk [87].

## 8. Future Research Possibilities

Even though the mechanisms behind the excessive cardiovascular risk in RA patients are partially explored, there are still aspects that can be further investigated. The most relevant ones include improvements in risk assessment. Future research can be focused on the possible implications of various biomarkers in risk stratification (for example, sCD163, humanin, and ischemia-modified albumin). In addition, mechanisms of regulation of these biomarkers can be studied in order to lower the cardiovascular risk and discover new therapy targets. Another prospective research idea could focus on the role of artificial intelligence in cardiovascular risk evaluation in RA patients.

## 9. Conclusions

In the context of traditional cardiovascular risk factors, RA-specific inflammatory and autoimmune mechanisms increase the risk of cardiovascular morbidity and mortality in this group of patients. Cardiovascular risk assessment in patients with RA should be performed regularly, taking into account the impact that persistent inflammation has on the atherosclerosis process. Ultrasound assessment of atherosclerotic plaques allows for an accurate classification of patients with RA into risk groups, facilitating the administration of an appropriate lipid-lowering treatment and a reduction in the risk of future CV events.

## Figures and Tables

**Table 1:** Lipid-lowering treatment according to the SCORE risk ([87] modified).

Recommended Lipid-Lowering Treatment	Risk Category—SCORE
No treatment	Risk < 5%
Considered treatment	Risk 5–<10% and LDL cholesterol ≥ 2.5 mmol/L (100 mg/dL)
Recommended treatment	Risk ≥ 10% and LDL cholesterol ≥ 1.8 mmol/L (70 mg/dL) and/or diagnosed cardiovascular disease

## Data Availability

No new data were created or analyzed in this study.

## Abbreviations

The following abbreviations are used in this manuscript:

ACC/AHA	American College of Cardiology/American Heart Association
ACCP	Anti-cyclic citrullinated peptide antibodies
ACR	American College of Rheumatology
ACS	Acute coronary syndrome
AF	Atrial fibrillation
ApoA-I	Apolipoprotein A-I
ApoB	Apolipoprotein B
AS	Aortic stenosis
ASA	Acetylsalicylic acid
AUC	Area under the curve
BAFF	B-cell activating factor
BMI	Body mass index
CCL-7 (MCP3)	Monocyte chemotactic protein 3
CDAI	Clinical disease activity index (for rheumatoid arthritis)
CD163	Cluster of differentiation 163
CHR	High-sensitive CRP:HDL-cholesterol ratio
CI	Confidence interval
CRP	C-reactive protein
CS	Corticosteroids
CV	Cardiovascular
CVD	Cardiovascular disease
CVR	Cardiovascular risk
DAS28	Disease activity score 28 for rheumatoid arthritis
DMARD	Disease-modifying anti-rheumatic drug
ED	Endothelial dysfunction
ELAM-1	Endothelial leukocyte adhesion molecule-1
ERS-RA	The expanded risk score in rheumatoid arthritis
ESC	European Society of Cardiology
ESR	Erythrocyte sedimentation rate
FRS	Framingham risk score
HAQ	Health assessment questionnaire
HAQ-DI	Health assessment questionnaire disability index
HDL	High-density lipoprotein
HDL2-C	HDL fraction 2
HDL3-C	HDL fraction 3
HF	Heart failure
HFpEF	Heart failure with preserved ejection fraction
HLA-DR1	Human leukocyte antigen DR-1
HIV	Human immunodeficiency virus
HR	Hazard ratio
ICAM-1	Inter-cellular adhesion molecule 1
IL-1,6,8,17	Interleukin 1, 6, 8, 17
IS	Ischemic stroke
LDL	Low-density lipoprotein
IMA	Ischemia-modified albumin
IMT	Intima–media thickness
MACE	Major adverse cardiovascular events
MCP	Metacarpophalangeal joint
METS-VF	Metabolic score for visceral fat
MFR	Myocardial flow reserve
MHC	Major histocompatibility complex
MI	Myocardial infarction
NET	Neutrophil extra-cellular trap
NSAID	Non-steroidal anti-inflammatory drug
OA	Osteoarthritis
OR	Odds ratio
QRISK2,3	Cardiovascular risk score
PAD	Peripheral artery disease
RA	Rheumatoid arthritis
RF	Rheumatoid factor
ROC	Receiver operating characteristic
RS	Reynolds risk score
RSN	Reactive species of nitrogen
RSO	Reactive species of oxygen
RV	Rheumatoid vasculitis
SAVR	Surgical aortic valve replacement
sCD163	Soluble cluster of differentiation 163
SCORE	Systematic COronary Risk Evaluation
SR-B1	Scavenger receptor, class B type 1
T2DM	Type 2 diabetes mellitus
TAVR	Transcatheter aortic valve replacement
TC	Total cholesterol
TG	triglycerides
TNF-α	Tumor necrosis factor-α
VCAM-1	Vascular cell adhesion molecule 1

## References

[1] Drosos A.A., Venetsanopoulou A.A., Pelechas E., Voulgari P.V. (2024). Exploring Cardiovascular Risk Factors and Atherosclerosis in Rheumatoid Arthritis. Eur. J. Intern. Med..

[2] Bedeković D., Bošnjak I., Bilić-Ćurčić I., Kirner D., Šarić S., Novak S. (2024). Risk for cardiovascular disease development in rheumatoid arthritis. BMC Cardiovasc. Disord..

[3] Murphy L., Saab M.M., Cornally N., McHugh S., Cotter P. (2024). Cardiovascular disease risk assessment in patients with rheumatoid arthritis: A scoping review. Clin. Rheumatol..

[4] Hannawi S., Hannawi H., Al Salmi I. (2020). Cardiovascular disease and subclinical atherosclerosis in rheumatoid arthritis. Hypertens. Res..

[5] Rohrich D.C., van de Wetering E.H.M., Rennings A.J., Arts E.E., Meek I.L., den Broeder A.A., Fransen J., Popa C.D. (2021). Younger age and female gender are determinants of underestimated cardiovascular risk in rheumatoid arthritis patients: A prospective cohort study. Arthritis Res. Ther..

[6] Hippisley-Cox J., Coupland C., Brindle P. (2017). Development and validation of QRISK3 risk prediction algorithms to estimate future risk of cardiovascular disease: Prospective cohort study. BMJ.

[7] Kattamuri L., Duggal S., Aparece J.P., Sairam S. (2025). Cardiovascular Risk Factor and Atherosclerosis in Rheumatoid Arthritis (RA). Curr. Cardiol. Rep..

[8] Arts E., Fransen J., Lemmers H., Stalenhoef A., Joosten L., van Riel P., Popa C.D. (2012). High-density lipoprotein cholesterol subfractions HDL2 and HDL3 are reduced in women with rheumatoid arthritis and may augment the cardiovascular risk of women with RA: A cross-sectional study. Arthritis Res. Ther..

[9] Chang K., Yang S.M., Kim S.H., Han K.H., Park S.J., Shin J.I. (2014). Smoking and rheumatoid arthritis. Int. J. Mol. Sci..

[10] Li J., Chen Y., Liu Q., Tian Z., Zhang Y. (2023). Mechanistic and therapeutic links between rheumatoid arthritis and diabetes mellitus. Clin. Exp. Med..

[11] Ruscitti P., Ursini F., Cipriani P., Liakouli V., Carubbi F., Berardicurti O., Sarro G.D., Giacomelli R. (2017). Poor clinical response in rheumatoid arthritis is the main risk factor for diabetes development in the shortterm: A 1-year, single-centre, longitudinal study. PLoS ONE.

[12] Otsuka Y., Kiyohara C., Kashiwado Y., Sawabe T., Nagano S., Kimoto Y., Ayano M., Mitoma H., Akahoshi M., Arinobu Y. (2018). Effects of tumor necrosis factor inhibitors and tocilizumab on the glycosylated hemoglobin levels in patients with rheumatoid arthritis; an observational study. PLoS ONE.

[13] Antohe J.L., Bili A., Sartorius J.A., Kirchner H.L., Morris S.J., Dancea S., Wasko M.C.M. (2012). Diabetes mellitus risk in rheumatoid arthritis: Reduced incidence with anti-tumor necrosis factor α therapy. Arthritis Care Res..

[14] Lu M.C., Yan S.T., Yin W.Y., Koo M., Lai N.S. (2014). Risk of rheumatoid arthritis in patients with type 2 diabetes: A nationwide population-based case-control study. PLoS ONE.

[15] Di Muzio C., Cipriani P., Ruscitti P. (2022). Rheumatoid Arthritis Treatment Options and Type 2 Diabetes: Unravelling the Association. BioDrugs.

[16] Zhang K., Jia Y., Wang R., Guo D., Yang P., Sun L., Wang F., Liu F., Zang Y., Shi M. (2023). Rheumatoid arthritis and the risk of major cardiometabolic diseases: A Mendelian randomization study. Scand. J. Rheumatol..

[17] Tang H., Zhao J., Yan X., Zheng Z., Bai W., Tang Z., Liu X. (2025). Causal Relationships Between Abdominal Obesity, Type 2 Diabetes, Fasting Insulin, and Cervical Disc Disorders, Osteoporosis, and Rheumatoid Arthritis: A Mendelian Randomization Study. J. Multidiscip. Healthc..

[18] Almalki Z.S., AlOmari B.A., Alshammari T., Alshlowi A., Khan M.F., Hazazi A., Alruwaily M., Alsubaie S., Alanazi F., Aldossary N. (2022). Uncontrolled blood pressure among hypertensive adults with rheumatoid arthritis in Saudi Arabia: A cross-sectional study. Medicine.

[19] Lauper K., Gabay C. (2017). Cardiovascular risk in patients with rheumatoid arthritis. Semin. Immunopathol..

[20] Erlandsson M.C., Lyngfelt L., Åberg N.D., Wasén C., Espino R.A., Silfverswärd S.T., Nadali M., Jood K., Andersson K.M.E., Pullerits R. (2019). Low serum IGF1 is associated with hypertension and predicts early cardiovascular events in women with rheumatoid arthritis. BMC Med..

[21] Marchand N.E., Sparks J.A., Tedeschi S.K., Malspeis S., Costenbader K.H., Karlson E.W., Lu B. (2021). Abdominal Obesity in Comparison with General Obesity and Risk of Developing Rheumatoid Arthritis in Women. J. Rheumatol..

[22] Li Y., Zhu Y., Tang X., Guo Z., Li J., Lv S., Liu M., Yu Y., Lei C. (2025). Association of visceral fat metabolism score with risk of rheumatoid arthritis in US adults. Front. Nutr..

[23] Passot C., Mulleman D., Bejan-Angoulvant T., Aubourg A., Willot S., Lecomte T., Picon L., Goupille P., Paintaud G., Ternant D. (2016). The underlying inflammatory chronic disease influences infliximab pharmacokinetics. MAbs.

[24] Lumeng C.N., Saltiel A.R. (2011). Inflammatory links between obesity and metabolic disease. J. Clin. Investig..

[25] Van der Helm-van Mil A.H., van der Kooij S.M., Allaart C.F., Toes R.E., Huizinga T.W. (2008). A high body mass index has a protective effect on the amount of joint destruction in small joints in early rheumatoid arthritis. Ann. Rheum. Dis..

[26] Muñoz-Barrera L., Perez-Sanchez C., Ortega-Castro R., Corrales S., Luque-Tevar M., Cerdó T., Sanchez-Pareja I., Font P., López-Mejías R., Calvo J. (2024). Personalized cardiovascular risk assessment in Rheumatoid Arthritis patients using circulating molecular profiles and their modulation by TNFi, IL6Ri, and JAKinibs. Biomed. Pharmacother..

[27] Kassem E., Ghonimy R., Adel M., El-Sharnoby G. (2011). Non traditional risk factors for carotid atherosclerosis in rheumatoid arthritis. Egypt. Rheumatol..

[28] Goodson N.J., Symmons D.P.M., Scott D.G.I., Bunn D., Lunt M., Silman A.J. (2005). Baseline levels of C-reactive protein and prediction of death from cardiovascular disease in patients with inflammatory polyarthritis: A ten year follow up study of a primary care-based inception cohort. Arthritis Rheum..

[29] Erre G.L., Cacciapaglia F., Sakellariou G., Manfredi A., Bartoloni E., Viapiana O., Fornaro M., Cauli A., Mangoni A.A., Woodman R.J. (2022). “Cardiovascular, Obesity and Rheumatic Disease Study (CORDIS) Group” of the Italian Society of Rheumatology (SIR). C-reactive protein and 10-year cardiovascular risk in rheumatoid arthritis. Eur. J. Intern. Med..

[30] Solomon D.H., Greenberg J., Curtis J.R., Liu M., Farkouh M.E., Tsao P., Kremer J.M., Etzel C.J. (2015). Derivation and internal validation of an expanded cardiovascular risk prediction score for rheumatoid arthritis: A Consortium of Rheumatology Researchers of North America Registry study. Arthritis Rheumatol..

[31] Roghani S.A., Shamsi A., Jalili C., Jalili F., Lotfi R., Garman N., Rostampour R., Taghadosi M. (2024). Interleukin-6 positively correlates with cardiovascular disease predictor algorithms and biomarker in rheumatoid arthritis patients. J. Cell. Mol. Med..

[32] Gerasimova E.V., Popkova T.V., Kirillova I.G., Gerasimova D.A., Nasonov E.L., Lila A.M. (2024). Interleukin-6: Cardiovascular Aspects of Long-Term Cytokine Suppression in Patients with Rheumatoid Arthritis. Int. J. Mol. Sci..

[33] Myasoedova E., Chandran A., Ilhan B., Major B.T., Michet C.J., Matteson E.L., Crowson C.S. (2016). The role of rheumatoid arthritis (RA) flare and cumulative burden of RA severity in the risk of cardiovascular disease. Ann. Rheum. Dis..

[34] Wells G., Becker J.C., Teng J., Dougados M., Schiff M., Smolen J., Aletaha D., van Riel P.L.C.M. (2009). Validation of the 28-joint Disease Activity Score (DAS28) and European League Against Rheumatism response criteria based on C-reactive protein against disease progression in patients with rheumatoid arthritis, and comparison with the DAS28 based on erythrocyte sedimentation rate. Ann. Rheum. Dis..

[35] Zaragoza-García O., Briceño O., Villafan-Bernal J.R., Gutiérrez-Pérez I.A., Rojas-Delgado H.U., Alonso-Silverio G.A., Alarcón-Paredes A., Navarro-Zarza J.E., Morales-Martínez C., Rodríguez-García R. (2025). Levels of sCD163 in women rheumatoid arthritis: Relationship with cardiovascular risk markers. Clin. Investig. Arterioscler..

[36] Greisen S.R., Moller H.J., Stengaard-Pedersen K., Hetland M.L., Hørslev-Petersen K., Jørgensen A., Deleuran B. (2011). Soluble macrophage-derived CD163 is a marker of disease activity and progression in early rheumatoid arthritis. Clin. Exp. Rheumatol..

[37] Coradduzza D., Cruciani S., Di Lorenzo B., De Miglio M.R., Zinellu A., Maioli M., Medici S., Erre G.L., Carru C. (2025). Plasma Humanin and Non-Coding RNAs as Biomarkers of Endothelial Dysfunction in Rheumatoid Arthritis: A Pilot Study. Non-Coding RNA.

[38] Dessein P.H., Joffe B.I., Singh S. (2005). Biomarkers of endothelial dysfunction, cardiovascular risk factors and atherosclerosis in rheumatoid arthritis. Arthritis Res. Ther..

[39] Erre G.L., Chessa I., Bassu S., Cavagna L., Carru C., Pintus G., Giordo R., Mangoni A.A., Sanna G.D., Zinellu A. (2024). Association between ischemia-modified albumin (IMA) and peripheral endothelial dysfunction in rheumatoid arthritis patients. Sci. Rep..

[40] Ram Chander S., Varikasuvu S.R., Anil Kumar P., Rupanagudi A. (2017). Ischemia modified albumin concentrations in patients with rheumatoid arthritis. Int. J. Rheum. Dis..

[41] Leitemperguer M.R., Tatsch E., Kober H., Moresco R.N. (2014). Assessment of ischemia-modified albumin levels in patients with rheumatoid arthritis. Clin. Lab..

[42] Pan S.Y., Tian H.M., Zhu Y., Gu W.J., Zou H., Wu X.Q., Cheng R.J., Yang Z. (2022). Cardiac damage in autoimmune diseases: Target organ involvement that cannot be ignored. Front. Immunol..

[43] Moran C.A., Collins L.F., Beydoun N., Mehta P.K., Fatade Y., Isiadinso I., Lewis T.T., Weber B., Goldstein J., Ofotokun I. (2022). Cardiovascular Implications of Immune Disorders in Women. Circ. Res..

[44] Gao C., Hou Q., Cao H., Li C., Peng X., Han Q., Wu S., Li K. (2025). Aspirin does not confer protection against major ischemic vascular events in patients diagnosed with rheumatoid arthritis. J. Int. Med. Res..

[45] Holmqvist M., Ljung L., Askling J. (2017). Acute coronary syndrome in new-onset rheumatoid arthritis: A population-based nationwide cohort study of time trends in risks and excess risks. Ann. Rheum. Dis..

[46] McCoy S.S., Crowson C.S., Maradit-Kremers H., Therneau T.M., Roger V.L., Matteson E.L., Gabriel S.E. (2013). Longterm outcomes and treatment after myocardial infarction in patients with rheumatoid arthritis. J Rheumatol..

[47] Rus M., Ardelean A.I., Judea Pusta C., Crisan S., Marian P., Pobirci L.O., Huplea V., Osiceanu A.S., Osiceanu G.A., Andronie-Cioara F.L. (2023). Prevalence of Cardiovascular Comorbidities in Patients with Rheumatoid Arthritis. Medicina.

[48] Kerola A.M., Ikdahl E., Engebretsen I., Bugge C., Semb A.G. (2024). Rheumatoid arthritis and the risk of ischaemic stroke after diagnosis of atrial fibrillation: A Norwegian nationwide register study. Rheumatology.

[49] Alqudah Q., Alomari A., Daise M., Awad A., Rhabneh L., Obeidat O., Obeidat O., Alomari S. (2025). Rheumatoid Arthritis and Atrial Fibrillation: A Complex Cardiovascular Intersection—Insights from a Retrospective Cohort Study. Med. Arch..

[50] Nadareishvili Z., Michaud K., Hallenbeck J.M., Wolfe F. (2008). Cardiovascular, Rheumatologic, and Pharmacologic Predictors of Stroke in Patients with Rheumatoid Arthritis: A Nested, Case–Control Study. Arthritis Rheum..

[51] Chen Y.R., Hsieh F.I., Lien L.M., Hu C.J., Jeng J.S., Peng G.S., Tang S.C., Chi A.-F., Sung Y.-F., Chiou H.-Y. (2018). Rheumatoid arthritis significantly increased recurrence risk after ischemic stroke/transient ischemic attack. J. Neurol..

[52] Wolfe F., Michaud K. (2004). Heart failure in rheumatoid arthritis: Rates, predictors, and the effect of anti-tumor necrosis factor therapy. Am. J. Med..

[53] Mantel Ä., Holmqvist M., Andersson D.C., Lund L.H., Askling J. (2017). Association Between Rheumatoid Arthritis and Risk of Ischemic and Nonischemic Heart Failure. J. Am. Coll. Cardiol..

[54] Løgstrup B.B., Ellingsen T., Pedersen A.B., Kjaersgaard A., Bøtker H.E., Maeng M. (2018). Development of heart failure in patients with rheumatoid arthritis: A Danish population-based study. Eur. J. Clin. Inv..

[55] Davis J.M., Roger V.L., Crowson C.S., Kremers H.M., Therneau T.M., Gabriel S.E. (2008). The presentation and outcome of heart failure in patients with rheumatoid arthritis differs from that in the general population. Arthritis Rheum..

[56] Bois J.P., Crowson C.S., Khullar T., Achenbach S.J., Krause M.L., Mankad R. (2017). Progression rate of severity of aortic stenosis in patients with rheumatoid arthritis. Echocardiography.

[57] Roldan C.A., DeLong C., Qualls C.R., Crawford M.H. (2007). Characterization of valvular heart disease in rheumatoid arthritis by transesophageal echocardiography and clinical correlates. Am. J. Cardiol..

[58] Wang Z., Lv J., Qian X., Li Z., Yin Z., Wang C., Zhao S., Gao X., Wu Y. (2025). Association Between Rheumatoid Arthritis and the Risk of Incident Degenerative Valvular Heart Disease: Evidence From a Prospective Cohort Study. J. Am. Heart Assoc..

[59] Peng B., Zha L., Juaiti M., Lin W., Zhou X., Ou Z., Zhang M., Yu Z., Tang Y. (2025). Association Between Inflammatory Arthritis, Genetic Risk, and the Long-Term Risk of Degenerative Aortic Stenosis: A Prospective Cohort Study. J. Am. Heart Assoc..

[60] Phua K., Chew N.W., Kong W.K., Tan R.S., Ye L., Poh K.K. (2022). The mechanistic pathways of oxidative stress in aortic stenosis and clinical implications. Theranostics.

[61] Johnson T.M., Mahabir C.A., Yang Y., Roul P., Goldsweig A.M., Binstadt B.A., Baker J.F., Sauer B.C., Cannon G.W., Mikuls T.R. (2023). Aortic Stenosis Risk in Rheumatoid Arthritis. JAMA Intern. Med..

[62] Kim A.R., Ji E., Lee S., Hong S., Kim D.H., Song J.M., Kang D.H., Song J.K. (2023). Association of citrullination with the progression of aortic stenosis. Sci. Rep..

[63] Chuang Y.W., Yu M.C., Lin C.L., Yu T.M., Shu K.H., Huang S.T., Kao C.H. (2016). Risk of peripheral arterial occlusive disease in patients with rheumatoid arthritis. A nationwide population-based cohort study. Thromb. Haemost..

[64] Zoubi T., Gordon H. (2023). Systematic review of associations between concomitant rheumatoid arthritis and peripheral arterial disease, health-related quality of life and functional capacity. Rheumatol. Int..

[65] Brevetti G., Giugliano G., Brevetti L., Hiatt W.R. (2010). Inflammation in Peripheral Artery Disease. Circulation.

[66] Sedrakyan S., Fatima T., Khatun M.K., Awan M.R., Okam N.A., Jahan N. (2020). Evaluation of the Risk of Getting Peripheral Artery Disease in Rheumatoid Arthritis and the Selection of Appropriate Diagnostic Methods. Cureu.

[67] Kishore S., Maher L., Majithia V. (2017). Rheumatoid Vasculitis: A Diminishing Yet Devastating Menace. Curr. Rheumatol. Rep..

[68] Makol A., Crowson C.S., Wetter D.A., Sokumbi O., Matteson E.L., Warrington K.J. (2014). Vasculitis associated with rheumatoid arthritis: A case-control study. Rheumatology.

[69] Laskari K., Ahmadi-Simab K., Lamken M., Csernok E., Gross W.L., Hellmich B. (2010). Are anti-cyclic citrullinated peptide autoantibodies seromarkers for rheumatoid vasculitis in a cohort of patients with systemic vasculitis?. Ann. Rheum. Dis..

[70] Turesson C., Schaid D.J., Weyand C.M., Jacobsson L.T., Goronzy J.J., Petersson I.F., Dechant S.A., Nyähll-Wåhlin B.M., Truedsson L., Sturfelt G. (2006). Association of HLA-C3 and smoking with vasculitis in patients with rheumatoid arthritis. Arthritis Rheum..

[71] Turesson C., Schaid D.J., Weyand C.M., Jacobsson L.T., Goronzy J.J., Petersson I.F., Sturfelt G., Nyhäll-Wåhlin B.M., Truedsson L., Dechant S.A. (2005). The impact of HLA-DRB1 genes on extra-articular disease manifestations in rheumatoid arthritis. Arthritis Res. Ther..

[72] Weber B., Weisenfeld D., Massarotti E., Seyok T., Cremone G., Lam E., Golnik C., Brownmiller S., Liu F., Huang S. (2024). Interplay Between Systemic Inflammation, Myocardial Injury, and Coronary Microvascular Dysfunction in Rheumatoid Arthritis: Results from the LiiRA Study. J. Am. Heart Assoc..

[73] Amigues I., Russo C., Giles J.T., Tugcu A., Weinberg R., Bokhari S., Bathon J.M. (2019). Myocardial Microvascular Dysfunction in Rheumatoid ArthritisQuantitation by 13N-Ammonia Positron Emission Tomography/Computed Tomography. Circ. Cardiovasc. Imaging.

[74] Liao K.P., Huang J., He Z., Cremone G., Lam E., Hainer J.M., Morgan V., Bibbo C., Carli M.D. (2021). Coronary Microvascular Dysfunction in Rheumatoid Arthritis Compared to Diabetes Mellitus and Association with All-Cause Mortality. Arthritis Car. Res..

[75] Ljung L., Ueda P., Liao K.P., Greenberg J.D., Etzel C.J., Solomon D.H., Askling J. (2018). Performance of the Expanded Cardiovascular Risk Prediction Score for Rheumatoid Arthritis in a geographically distant National Register-based cohort: An external validation. RMD Open.

[76] Kellner H., Bornholdt K., Hein G. (2010). Leflunomide in the treatment of patients with early rheumatoid arthritis--results of a prospective non-interventional study. Clin. Rheumatol..

[77] Visseren F.L.J., Mach F., Smulders Y.M., Carballo D., Koskinas K.C., Bäck M., Benetos A., Biffi A., Boavida J.-M., Capodanno D. (2021). 2021 ESC Guidelines on cardiovascular disease prevention in clinical practice. Eur. Heart J..

[78] Crowson C.S., Matteson E.L., Roger V.L., Therneau T.M., Gabriel S.E. (2012). Usefulness of risk scores to estimate the risk of cardiovascular disease in patients with rheumatoid arthritis. Am. J. Cardiol..

[79] Ridker P.M., Paynter N.P., Rifai N., Gaziano J.M., Cook N.R. (2008). C-reactive protein and parental history improve global cardiovascular risk prediction: The Reynolds Risk Score for men. Circulation.

[80] Arts E.E., Popa C., Den Broeder A.A., Semb A.G., Toms T., Kitas G.D., van Riel P.L., Fransen J. (2015). Performance of four current risk algorithms in predicting cardiovascular events in patients with early rheumatoid arthritis. Ann. Rheum. Dis..

[81] Colaco K., Ocampo V., Ayala A.P., Harvey P., Gladman D.D., Piguet V., Eder L. (2020). Predictive Utility of Cardiovascular Risk Prediction Algorithms in Inflammatory Rheumatic Diseases: A Systematic Review. J. Rheumatol..

[82] Arts E.E., Popa C.D., Den Broeder A.A., Donders R., Sandoo A., Toms T., Rollefstad S., Ikdahl E., Semb A.G., Kitas G.D. (2016). Prediction of cardiovascular risk in rheumatoid arthritis: Performance of original and adapted SCORE algorithms. Ann. Rheum. Dis..

[83] Wahlin B., Innala L., Magnusson S., Möller B., Smedby T., Rantapää-Dahlqvist S., Wållberg-Jonsson S. (2019). Performance of the Expanded Cardiovascular Risk Prediction Score for Rheumatoid Arthritis Is Not Superior to the ACC/AHA Risk Calculator. J. Rheumatol..

[84] Goff D.C., Lloyd-Jones D.M., Bennett G., Coady S., D’agostino R.B., Gibbons R., Greenland P., Lackland D.T., Levy D., O’Donnell C.J. (2014). 2013 ACC/AHA guideline on the assessment of cardiovascular risk: A report of the American College of Cardiology/American Heart Association Task Force on Practice Guidelines. Circulation.

[85] Kawai V.K., Chung C.P., Solus J.F., Oeser A., Raggi P., Stein C.M. (2015). The ability of the 2013 American College of Cardiology/American Heart Association cardiovascular risk score to identify rheumatoid arthritis patients with high coronary artery calcification scores. Arthritis Rheumatol..

[86] Agca R., Heslinga S.C., Rollefstad S., Helsinga M., McInnes I.B., Peters M.J.L., Kvien T.K., Dougados M., Radner H., Atzeni F. (2017). EULAR recommendations for cardiovascular disease risk management in patients with rheumatoid arthritis and other forms of inflammatory joint disorders: 2015/2016 update. Ann. Rheum. Dis..

[87] Semb A.G., Ikdahl E., Hisdal J., Olsen I.C., Rollefstad S. (2016). Exploring cardiovascular disease risk evaluation in patients with inflammatory joint diseases. Int. J. Cardiol..

[88] Del Rincón I., Williams K., Stern M.P., Freeman G.L., O’Leary D.H., Escalante A. (2003). Association between carotid atherosclerosis and markers of inflammation in rheumatoid arthritis patients and healthy subjects. Arthritis Rheum..

[89] González Mazarío R., Fragío Gil J.J., Martínez Calabuig P., Grau García E., Cañada Martínez A.J., Román Ivorra J.A. (2022). Cardiovascular risk assessment with carotid ultrasound in rheumatoid arthritis. Med. Clin..

[90] Skeoch S., Cristinacce P.L.H., Williams H., Pemberton P., Xu D., Sun J., James J., Yuan C., Hatsukami T., Hockings P.D. (2017). Imaging atherosclerosis in rheumatoid arthritis: Evidence for increased prevalence, altered phenotype and a link between systemic and localised plaque inflammation. Sci. Rep..

